# One‐Pot Synthesis of Chiral *N*‐Arylamines by Combining Biocatalytic Aminations with Buchwald–Hartwig *N*‐Arylation

**DOI:** 10.1002/anie.202006246

**Published:** 2020-08-11

**Authors:** Sebastian C. Cosgrove, Matthew P. Thompson, Syed T. Ahmed, Fabio Parmeggiani, Nicholas J. Turner

**Affiliations:** ^1^ Department of Chemistry University of Manchester Manchester Institute of Biotechnology 131 Princess Street Manchester M1 7DN UK; ^2^ Future Biomanufacturing Research Hub University of Manchester Manchester Institute of Biotechnology 131 Princess Street Manchester M1 7DN UK; ^3^ Department of Chemistry, Materials and Chemical Engineering “G. Natta” Politecnico di Milano Via Mancinelli 7 20131 Milano Italy; ^4^ Current address: EnginZyme AB Tomtebodavägen 6, House A1, Floor 4 17165 Solna Sweden

**Keywords:** amines, biocatalysis, chemo-enzymatic synthesis, enzymes, palladium catalysis

## Abstract

The combination of biocatalysis and chemo‐catalysis increasingly offers chemists access to more diverse chemical architectures. Here, we describe the combination of a toolbox of chiral‐amine‐producing biocatalysts with a Buchwald–Hartwig cross‐coupling reaction, affording a variety of α‐chiral aniline derivatives. The use of a surfactant allowed reactions to be performed sequentially in the same flask, preventing the palladium catalyst from being inhibited by the high concentrations of ammonia, salts, or buffers present in the aqueous media in most cases. The methodology was further extended by combining with a dual‐enzyme biocatalytic hydrogen‐borrowing cascade in one pot to allow for the conversion of a racemic alcohol to a chiral aniline.

## Graphical abstract

A two‐step, one‐pot chemoenzymatic synthesis of chiral anilines is reported. Chiral‐amine‐producing biocatalysts have been combined in the same pot as a Pd catalyst to allow the direct conversion of prochiral and racemic starting materials to enantiopure anilines. The use of a surfactant to enable this one‐pot process is a first for biocatalysis.

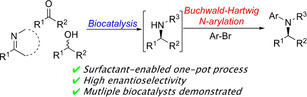

*N*‐arylamines are an important structural motif present in an array of bioactive molecules including APIs and agrochemicals (Figure 1). Methods for the synthesis of these compounds in enantiomerically enriched form, that are typically based on asymmetric reductive aminations of ketones using anilines as the amine donor, are well known.^1^ Transition metal catalysed reductive amination often requires elevated temperatures and pressures in addition to complex chiral ligands to achieve satisfactory yields and enantiomeric excess (*ee*).^2^, ^3^, ^4^, ^5^ On the other hand, organocatalytic approaches require anhydrous conditions in order to proceed however excellent yield and *ee* can be obtained under relatively mild conditions.^6^, ^7^, ^8^

**Figure 1:**
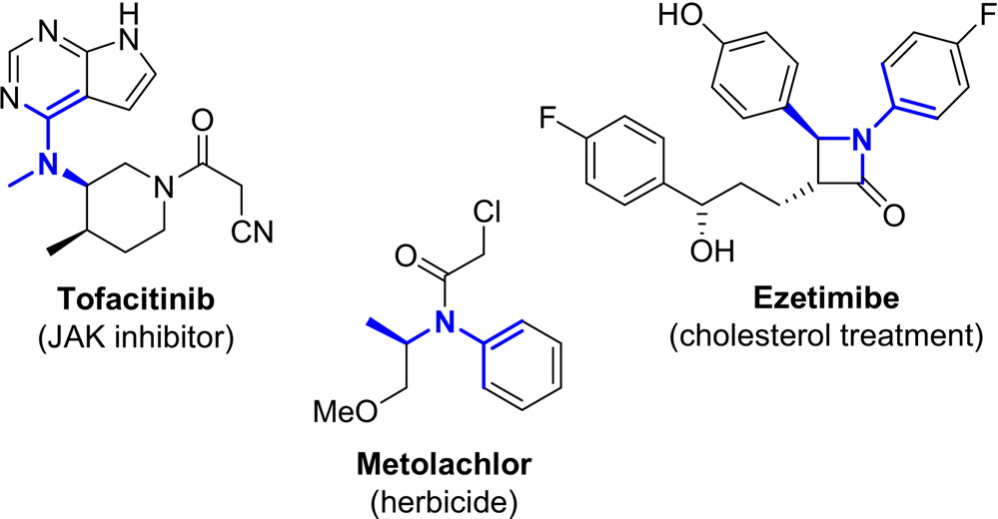
High‐value chiral *N*‐arylamine derivatives.

Biocatalysis represents a powerful tool for the synthesis of enantiomerically pure chiral amines.^9^ Several different enzyme classes have been reported including amine dehydrogenases (AmDH),^10^, ^11^ imine reductases (IRED) and reductive aminases (RedAm),^12^, ^13^, ^14^ amine oxidases (MAO),^15^, ^16^ ω‐transaminases (ω‐TA),^17^, ^18^ and engineered cytochrome P411s.^19^, ^20^ Despite the broad application of these biocatalysts for the synthesis of both cyclic and acyclic 1°, 2° and 3° amines, there have been few reports describing the application of these systems for the synthesis of *N*‐aryl amines.^15^, ^21^, ^22^ Interestingly, two recent papers reported homologues of *Asp*RedAm that accepted aniline as amine donor with conversions up to 99 %, however the substrate scope was limited.^23^, ^24^

The combination of biocatalysts and chemo‐catalysts allows chemists to effect overall transformations with high efficiency, not achievable using the individual catalysts alone.^25^, ^26^, ^27^, ^28^ Several strategies for merging precious‐metal catalysis and biocatalysis have been reported.^29^, ^30^, ^31^, ^32^, ^33^, ^34^ In particular, palladium catalysed methods have featured in several reported chemoenzymatic reactions, with biocatalytic processes coupled to Suzuki–Miyaura^35^ and Heck^36^ cross‐coupling or Wacker^37^ oxidation. One frequently used Pd‐catalysed transformation yet to be combined with biocatalysis is the Buchwald‐Hartwig cross‐coupling (BHA), which couples nucleophilic amines and aryl halides to afford anilines.^38^, ^39^, ^40^, ^41^

Herein we present a chemoenzymatic approach to chiral *N*‐arylamines, combining a variety of enantioselective biocatalytic transformations with a surfactant‐enabled BHA (Scheme 1).^11^, ^40^, ^42^

**Scheme 1:**
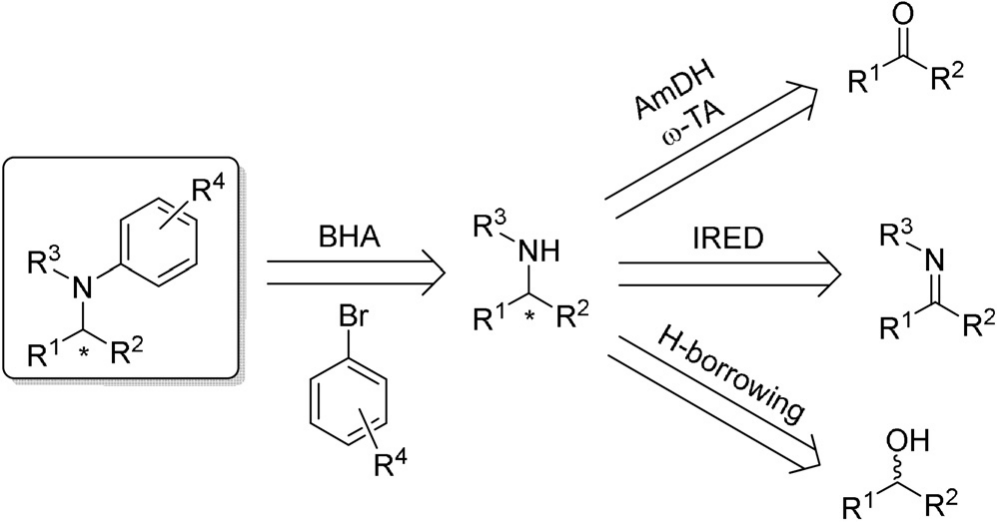
Retrosynthetic strategies for the chemoenzymatic formation of chiral *N*‐arylamines.

We initially envisaged a one‐pot process involving conversion of the substrate (ketone/imine/alcohol) followed by in situ BHA in an aqueous environment without any intermediate workup. In line with the principles of green chemistry there is significant interest in performing transition metal catalysed reactions in water.^43^ For example, the use of water‐soluble palladium sources in the Suzuki cross‐coupling combined with an alcohol dehydrogenase (ADH) reported by Borchert et al., gave excellent yields and *ee* but is limited to water soluble starting materials.^33^ Other approaches using heterogeneous, immobilised or compartmentalised catalysts have also been described.^29^, ^34^

One plausible reason for the limited number of one‐pot chemo‐enzymatic processes reported is the low solubility of many organic reagents in water,^44^ leading to slow reaction rates and low yields. The Lipshutz group have reported a designer surfactant DL‐α‐tocopherol methoxypolyethylene glycol succinate (TPGS‐750‐M, Figure 2) that enables a variety of important cross‐coupling reactions to proceed in water.^45^, ^46^, ^47^ Owing to the relatively low concentration of the surfactant, reactants are highly concentrated within the lipophilic core. Rate acceleration, even at room temperature, is commonly observed as a result. The Lipshutz group demonstrated recently that TPGS‐750‐M could be used in one‐pot, chemo‐enzymatic transformations using several precious‐metal catalysts and ADH enzymes.^48^ They showed Pd could be used in conjunction with ADH as well, however the biotransformation mixture was added after the metal‐catalysed step was complete.

**Figure 2:**
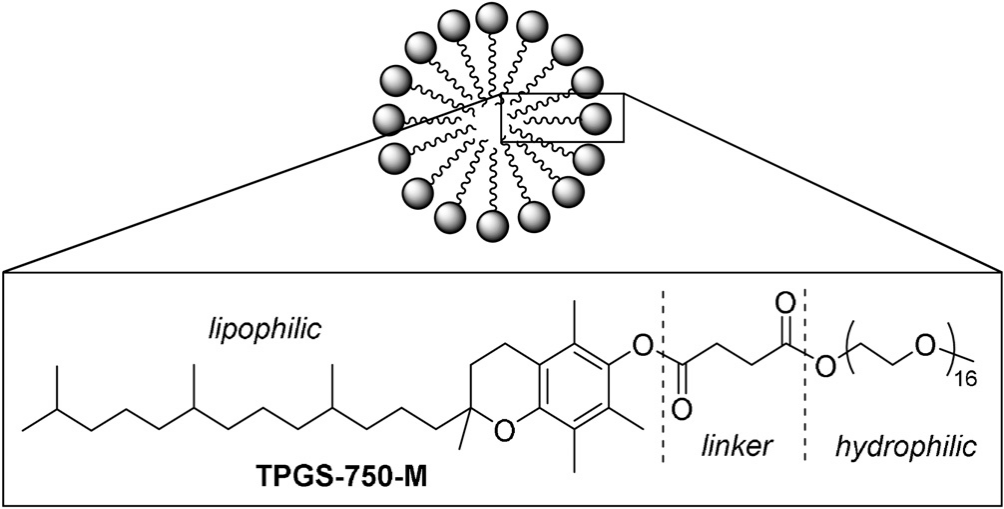
Structural components of TPGS‐750‐M.

From the outset, it was unknown if either the enzyme or any component of the biotransformation mixture would be detrimental to the performance of the cross‐coupling step. Initially we chose to use engineered amine dehydrogenases (AmDHs), which use ammonia as the sole amine source.^10^, ^42^ AmDHs have also been applied in an elegant redox‐neutral cascade alongside ADHs for the asymmetric amination of alcohols.^49^, ^50^, ^51^, ^52^ Hence, there is also an opportunity to generate optically enriched *N*‐arylamines from racemic alcohols.

Initially we screened a number of palladium pre‐catalysts, ligands and conditions to promote the coupling of model substrate *rac*‐**1** with bromobenzene **2 a** (Table 1).^45^, ^53^, ^54^ To investigate any potential inhibitory effects, the cross‐coupling conditions were screened in the presence of reagents required for the biocatalytic step. The impact of different biocatalyst preparations (cell free extract (CFE) or purified proteins), and the presence of high concentrations of ammonium salts were thus investigated.

**Table 1:** Optimisation of Buchwald‐Hartwig amination under biocatalyst conditions. 
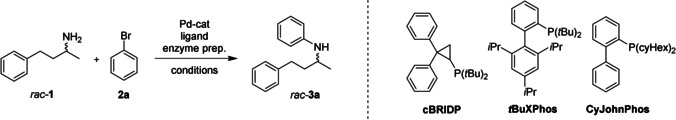

Entry	*T*	Solvent	Pd‐cat	Ligand	Additive	Conv. [%]^[a]^
1	100	water	[PdCl(cinnamyl)]_2_	CyJohnPhos	n.a.	6
2	100	1 m NH_4_Cl	[PdCl(cinnamyl)]_2_	CyJohnPhos	n.a.	0
3	room temp.	water	[PdCl(allyl)]_2_	cBRIDP	TPGS‐750‐M	57 (24 h)
4	room temp.	1 m NH_4_Cl	[PdCl(allyl)]_2_	cBRIDP	TPGS‐750‐M	26
5	40	1 m NH_4_Cl	[PdCl(allyl)]_2_	cBRIDP	TPGS‐750‐M	>99
6	40	1 m NH_4_Cl +CFE	[PdCl(allyl)]_2_	cBRIDP	TPGS‐750‐M	9
7	40	1 m NH_4_Cl+filtered CFE	[PdCl(allyl)]_2_	cBRIDP	TPGS‐750‐M	23
8	room temp.	water	[PdCl(allyl)]_2_	*t*BuXPhos	TPGS‐750‐M	0
9	50	water	[PdCl(allyl)]_2_	*t*BuXPhos	TPGS‐750‐M	>99
10	50	1 m NH_4_Cl	[PdCl(allyl)]_2_	*t*BuXPhos	TPGS‐750‐M	>99
11	50	1 m NH_4_Cl+CFE	[PdCl(allyl)]_2_	*t*BuXPhos	TPGS‐750‐M	80
12	50	1 m NH_4_Cl+filtered CFE	[PdCl(allyl)]_2_	*t*BuXPhos	TPGS‐750‐M	95
13	50	1 m NH_4_Cl+purified enzyme	[PdCl(allyl)]_2_	*t*BuXPhos	TPGS‐750‐M	90

[a] Conversion determined by GC‐FID analysis.

The ligand *t*BuXPhos, has been reported to be a general ligand suited to the coupling of primary amines,^54^ and in combination with the [PdCl(allyl)]_2_ pre‐catalyst reliably gave the best conversions in the absence of the biocatalyst. Importantly, the addition of concentrated ammonium chloride (necessary for the AmDH step) did not affect the outcome of the cross‐coupling (entry 10). Finally, it was noted that in the presence of the CFE containing the biocatalyst (entry 11) the conversions were lower than with filtered CFE (entry 12). However, in the presence of the purified enzymes the conversions were good to excellent (90 %) (entry 13). Encouraged that the chemoenzymatic process could be combined efficiently in a one‐pot, two‐step process, the reductive amination of ketones **4** and **7** (50 mm) to afford amines (*R*)‐**1** and (*R*)‐**5** (90 % conversion, 90 % and >99 % *ee*, respectively) was performed using previously optimised conditions for AmDH biotransformations.^55^ The crude reaction mixture was diluted 1:1 with a solution of TPGS‐750‐M (5 wt % in H_2_O) followed by addition of the reagents for the cross‐coupling step (Scheme 2).

**Scheme 2:**
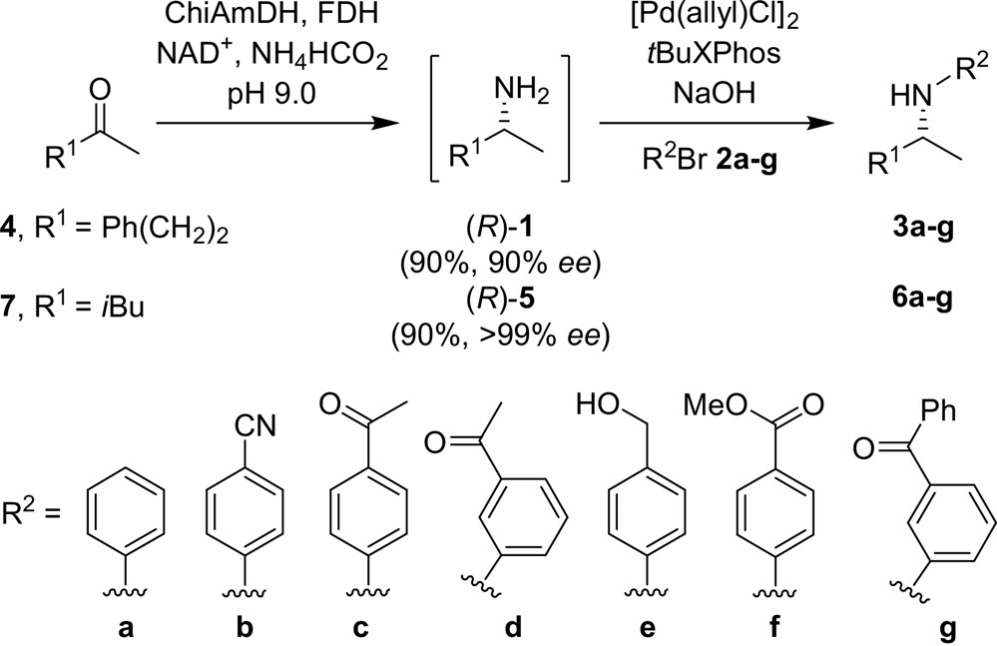
Combined AmDH amination with BHA of ketones **4** and **7**.

The coupling of amines (*R*)‐**1** and (*R*)‐**5** with a panel of aryl bromides **2 a**–**g** was accomplished using the optimised conditions for the cross‐coupling step to yield *N*‐arylamines **3 a**–**g** and **6 a**–**g** with good to excellent conversions (Table 2). Importantly, the chirality generated during the biocatalytic step was preserved during the cross‐coupling step (see Supporting Information).

**Table 2:** Conversions for *N*‐arylation of amines (*R*)‐**1** and (*R*)‐**5** with aryl bromides **2 a**‐**g**. 
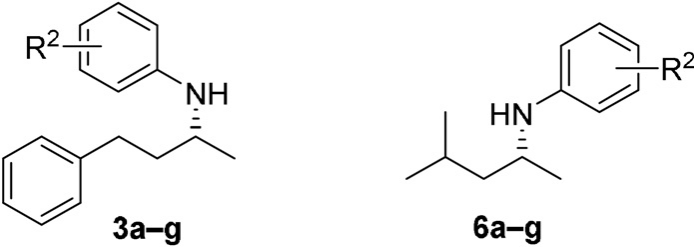

Product	Conv. (%)^[a,b]^	Product	Conv. (%)^[a,b]^
**3 a**	90 (81)	**6 a**	90 (81)
**3 b**	71 (64)	**6 b**	65 (59)
**3 c**	93 (83)	**6 c**	82 (74)
**3 d**	91 (82)	**6 d**	81 (73)
**3 e**	62 (56)	**6 e**	59^[c]^ (53)
**3 f**	70 (63)	**6 f**	54 (49)
**3 g**	76 (68)	**6 g**	90 (81)

[a] Conversion determined by GC‐FID analysis. [b] Conversion for Buchwald‐Hartwig step indicated out of brackets, with overall conversion from ketone to aniline indicated in brackets. [c] Oxidation to the corresponding aromatic aldehyde was observed.

We next considered a one‐pot *N*‐arylation reaction in which the chiral amine was generated by an ω‐transaminase (ω‐TA).^17^ ω‐TAs are attractive options for the first step since they possess broad substrate scope and give excellent *ee*s.^17^, ^18^ The transamination of ketone **4** (50 mm) with ATA‐117 (Codexis, USA) gave (*R*)‐**1** (>99 % conversion, >99 % *ee*) using an excess of alanine as the amine donor. However, when the one‐pot *N*‐arylation of **1** with **2 a** was attempted, no coupling was observed, presumably due to the presence of the competing nucleophilic amine alanine. Instead, the reaction mixture from the biotransformation containing (*R*)‐**1** was extracted into toluene and then coupling of (*R*)‐**1** with **2 a**–**c** and **2 f**,**g** could be performed under reported conditions to give **3 a**–**c** and **3 f**,**g** (63–99 % conversion and >99 % *ee*) (see Supporting Information).

To further exemplify the scope of our combined chemo‐enzymatic approach, we envisaged extending the process to the *N*‐arylation of cyclic chiral amines to furnish tertiary *N*‐arylamines. Imine reductases (IREDs) have been reported for the reduction of cyclic imines,^22^ amongst which the (*S*)‐imine reductase (*S*‐IRED) from *Streptomyces* sp. GF3546 is reported to catalyse the asymmetric reduction of cyclic five‐, six‐, and seven‐membered imines.^12^, ^56^

The *S*‐IRED catalysed asymmetric reduction of pyrroline **8** was performed under previously reported conditions to afford (*S*)‐2‐methylpyrrolidine **9** (80 % conv. >92 % *ee*). The reaction mixture was then diluted with a solution of TPGS‐750‐M as described above. Using the Takasago ligand cBRIDP in lieu of *t*BuXPhos^57^ gave efficient coupling with aryl bromides **2 a**–**g** to the corresponding *N*‐arylamines **10 a**–**g** with good to excellent conversions (Table 3). To demonstrate the synthetic utility of this method, **10 a** was prepared on a preparative scale with the addition of a toluene layer for the BHA, affording >100 mg in a 65 % isolated yield and >99 % *ee* (see Supporting Information).

**Table 3:** Conversions for *N*‐arylation of amine (*S*)‐**9** with aryl bromides **2 a**–**g**. 
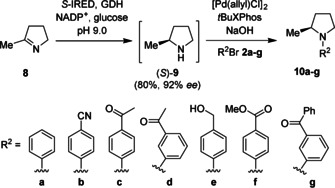

Product	Conv. (%)^[a,b]^
**10 a**	93 (74)
**10 b**	84 (67)
**10 c**	94 (75)
**10 d**	95 (76)
**10 e**	67 (54)
**10 f**	61 (49)
**10 g**	77 (62)

[a] Conversion determined by GC‐FID analysis. [b] Conversion for Buchwald‐Hartwig step indicated out of brackets, with overall conversion from ketone to aniline indicated in brackets.

Lastly, the same methodology was shown to be compatible with the recently developed biocatalytic hydrogen‐borrowing amination of alcohols.^52^ The system employs a non‐enantioselective variant (W110A‐G198D) of an alcohol dehydrogenase from *Thermoanaerobacter ethanolicus* coupled with ChiAmDH (Scheme 3). On a preparative scale the chiral *N*‐arylamine **6 c** was obtained directly from the racemic alcohol **11** with moderate yield and >99 % *ee*. The modest isolated yield of (*R*)‐**6 c** can be ascribed to some of the limitations of this methodology, namely: (i) the challenge of extraction and purification of products from a surfactant‐containing mixture, and (ii) the equilibrium‐based limitations to the conversion to the amine. Both issues could be addressed in the future through reaction engineering, for example in situ product removal could be applied to shift the equilibrium of the reaction, or crystallization could be used to improve isolation of products from the complex reaction mixture.

**Scheme 3:**
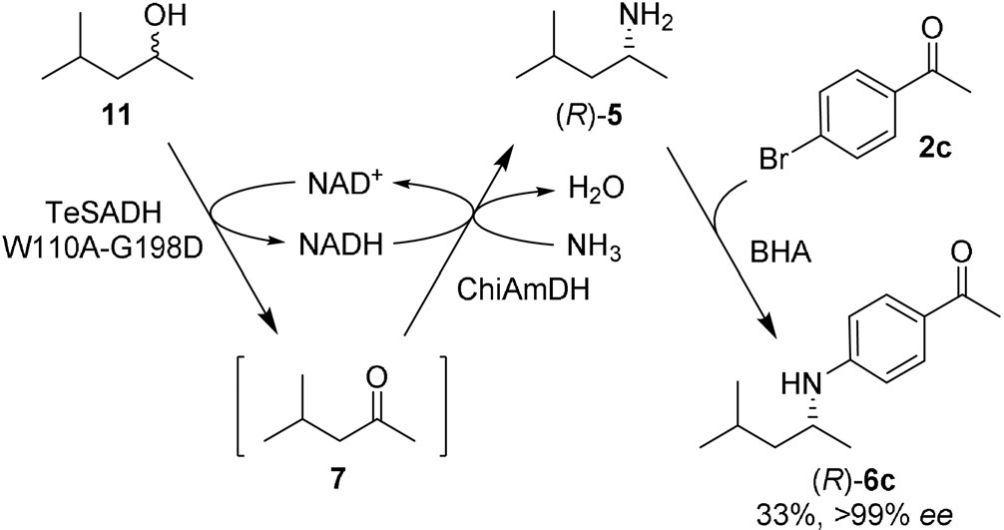
Hydrogen‐borrowing amination of **11**, followed by *N*‐arylation to afford (*R*)‐**6 c**.

In summary, we have demonstrated a new approach to the synthesis of chiral *N*‐arylamines by combining biocatalytic reductive amination or imine reduction with surfactant‐enabled Buchwald‐Hartwig cross‐coupling. Conversions to the corresponding *N*‐arylated amines are good to excellent (up to 90 %). Importantly, the asymmetric centre established in the biocatalytic step is unaffected by the subsequent cross‐coupling reaction.

We envisage that this biocompatible, surfactant‐enabled cross‐coupling approach will broaden the application of chemo‐enzymatic processes for the synthesis of high‐value compounds.

## Conflict of interest

The authors declare no conflict of interest.

## Supporting information

As a service to our authors and readers, this journal provides supporting information supplied by the authors. Such materials are peer reviewed and may be re‐organized for online delivery, but are not copy‐edited or typeset. Technical support issues arising from supporting information (other than missing files) should be addressed to the authors.

Supplementary
