# Supplementary material for: One‐Pot Synthesis of Chiral N‐Arylamines by Combining Biocatalytic Aminations with Buchwald–Hartwig N‐Arylation
Source: Angew Chem Int Ed Engl. 2020 Aug 11;59(41):18156–60. doi: 10.1002/anie.202006246 (PMC7590080; doi:10.1002/anie.202006246)
Supplement: Supplementary file 1 — Supplementary [file ANIE-59-18156-s001.pdf]

Angewandte Chemie
Eine Zeitschrift der Gesellschaft Deutscher Chemiker

Supporting Information

One-Pot Synthesis of Chiral *N*-Arylamines by Combining Biocatalytic Aminations with Buchwald–Hartwig *N*-Arylation

Sebastian C. Cosgrove[+], Matthew P. Thompson[+], Syed T. Ahmed, Fabio Parmeggiani, and Nicholas J. Turner**

anie_202006246_sm_miscellaneous_information.pdf

TABLE OF CONTENTS

Materials and General Methods **4**
General 4
General procedure for ChiAmDH catalysed reductive amination of ketones 5
General procedure for the one-pot *N*-arylation with ChiAmDH biotransformation mixture 5
General procedure for ATA-117 catalysed amination of ketones 5
General procedure for the two step *N*-arylation 5
General procedure for *S*-IRED catalysed reduction of imine 6
General procedure for the one-pot *N*-arylation with S-IRED biotransformation mixture 7
General procedure for the preparation *N*-arylated of amine standards 8
Analytics **17**
Method for determination of conversion: 17
Methods for determination of enantiomeric excess 18
GC-FID for determination of enantiomeric excess of (*R*)-1 20
GC-FID for determination of enantiomeric excess of (*R*)-5 21
GC-FID for determination of enantiomeric excess of (*S*)- 22
3a - 1H NMR 23
3a – 13C NMR 23
3b – 1H NMR 24
3b – 13C NMR 24
3c – 1H NMR 25
3c – 13C NMR 25
3d – 1H NMR 26
3d – 13C NMR 26
3e – 1H NMR 27
3e – 13C NMR 27
3f – 1H NMR 28
3f – 13C NMR 28
3g – 1H NMR 29
3g – 13C NMR 29
6a – 1H NMR 30
6a – 13C NMR 30
6b – 1H NMR 31
6b – 13C NMR 31
6c – 1H NMR 32
6c – 13C NMR 32
6d – 1H NMR 33
6d – 13C NMR 33
6e – 1H NMR 34
6e – 13C NMR 34
6f – 1H NMR 35
6f – 13C NMR 35
6g – 1H NMR 36
6g – 13C NMR 36
10a – 1H NMR 37
10a – 13C NMR 37

10b – 1H NMR 38
10b – 13C NMR 38
10c – 1H NMR 39
10c – 13C NMR 39
10d – 1H NMR 40
10d – 13C NMR 40
10e – 1H NMR 41
10e – 13C NMR 41
10f – 1H NMR 42
10f – 13C NMR 42
10g – 1H NMR 43
10g – 13C NMR 43
6c - 1H NMR from scaled up amination of 11 47
References 54

Materials and General Methods

General

^{1}H and ^{13}C NMR were recorded on a Bruker Avance 400 instrument (400 MHz for ^{1}H and 100 MHz for ^{13}C) in $CDCl_3$ using residual protic solvent as an internal standard. Reported chemical shifts (δ) (in parts per million (ppm) are relative to the residual protic solvent signal ($CHCl_3$ in $CDCl_3$, ^{1}H = 7.26; $CDCl_3$, ^{13}C = 77.0). GC analysis was performed on an Agilent 6850 GC (Agilent, Santa Clara, CA, USA) with a flame ionization detector (FID) and autosampler. HPLC was performed on an Agilent 1200 series system (Santa Clara, CA, USA) equipped with a G1379A degasser, G1312A binary pump, a G1367A well plate autosampler unit, a G1316A temperature-controlled column compartment and a G1315C diode array detector. Chromatograms were monitored at 265 nm. All solvent mixtures are given in (v/v) ratios. All chemicals were purchased from Sigma-Aldrich and were used as supplied.

The ChiAmDH and TeSADH, *cb*FDH and *S*-IRED were produced, purified and used as reported previously.[1–3] The ATA-117 (Codexis, USA) was supplied as a lyophilized powder that was solubilized in potassium phosphate buffer before use (50 mM, pH 8).

General procedure for ChiAmDH catalysed reductive amination of ketones

The reductive amination of ketones **4** and **7** was performed in ammonium formate buffer (final conc. 1M, pH 9.0, 20 mL), containing NAD (1 mM), purified ChiAmDH (1 mg/mL) and cbFDH (0.25 mg/mL) and DMSO (5 % v/v). The substrate (50 mM) was added directly and the reactions were shaken in a bench-top shaker at 37 °C. After 48h, small aliquots of the reactions were quenched by the addition of 10M NaOH (100 μL), and extracted with EtOAc (2 x 350 μL). The combined organics were dried over anhydrous $MgSO_4$. Conversion to amine and enantiomeric excess was analysed by GC-FID as detailed in the section "Analytics". The remainder of the reaction buffer was stored at 4 °C until use in the *N*-arylation reactions.

General procedure for the one-pot *N*-arylation with ChiAmDH biotransformation mixture

To a thoroughly degassed solution of TPGS-750-M (1 mL, 5 wt. % in H_2O) was added NaOH (4 mg, 100 μmol), *t*BuXPhos (2.5 mg, 6 μmol, 0.12 eq.) and palladium allyl chloride (1.8 mg, 5 μmol, 0.1 eq.). The solution was bubbled with nitrogen for 10 minutes followed by addition of the appropriate aryl bromide (0.08 mmol) and appropriate biotransformation mixture (1mL) containing amine **(*R*)-1** or **(*R*)-5** (ca. 50 μmol). Reactions were stirred at 50 °C for 24 hours and subsequently quenched by extraction with EtOAc (5 mL). The organics were dried over anhydrous $MgSO_4$. Conversion to and enantiomeric excess of the *N*-arylamine was determined as detailed in the section "Analytics"

General procedure for ATA-117 catalysed amination of ketones

The asymmetric amination of ketone **4** was performed in potassium phosphate buffer (50 mM, pH 8) containing ATA-117 (1 mg/mL), lactate dehydrogenase (1 mg/mL), PLP (1 mM), NAD+ (1 mM) and DMSO (5 % v/v) and D-alanine (250 mM) glucose dehydrogenase (1 mg/mL) and glucose (100 mM). The substrate (50 mM) was added directly and the reactions were shaken in a bench-top shaker at 30 °C. After 48h, small aliquots of the reactions were quenched by the addition of 10M NaOH (100 μL), and extracted with EtOAc (2 x 350 μL).
The combined organics were dried over anhydrous $MgSO_4$. Conversion to amine and enantiomeric excess was analysed by GC-FID as detailed in the section "Analytics" The remainder of the reaction buffer was stored at 4 °C until use in the *N*-arylation reactions.

General procedure for the two step *N*-arylation

The reaction mixture from the transaminase reaction (1 mL) containing amine **(*R*)-1** (50 μmol) was basified to pH 14 by addition of NaOH (100 μL) and extracted with toluene (3 x 2 mL). The combined organics were dried over anhydrous

$MgSO_4$.. The toluene solution was charged with NaOtBu (10 mg, 100 µmol, 2 eq.), CyJohnPhos (2 mg, 5 µmol, 0.1 eq.) and $Pd(OAc)_2$ (1.5 mg, 7 µmol, 0.14 eq.). The solution was bubbled with nitrogen for 10 minutes followed by addition of the appropriate aryl bromide (60 µmol, 1.2 eq.). Reactions were refluxed for 20 hours and subsequently quenched by addition of water (5 mL). The organics were dried over anhydrous $MgSO_4$. Conversion to and enantiomeric excess of the *N*-arylamine was determined as detailed in the section "Analytics". Conversions to the *N*-arylamines **3a-c** and **3f-g** are given in table S1.

Table S1 Conversions for two-step *N*-arylation from transaminase catalysed amination of (*R*)-1

Product	Conversion (%)
**3a**	99
**3b**	63
**3c**	88
**3f**	69
**3g**	98

a) Conversions determined by GC-FID

General procedure for *S*-IRED catalysed reduction of imine 8

The reduction of imine **8** was performed using potassium phosphate buffer (final conc. 100 mM, pH 8.0), containing NADP (1 mM), purified S-IRED (1 mg/mL) and DMSO (5 % v/v) The substrate (50 mM) was added directly and the reactions were shaken in a bench-top shaker at 30 °C. After 48h, small aliquots of the reactions were quenched by the addition of 10M NaOH (100 µL), and extracted with EtOAc (2 x 350 µL). The combined organics were dried over anhydrous $MgSO_4$. Conversion to amine **(*S*)-9** and enantiomeric excess was analysed by GC-FID as detailed in the section "Analytics" The remainder of the reaction buffer was stored at 4 °C until use in the arylation reactions.

General procedure for the one-pot *N*-arylation with *S*-IRED biotransformation mixture

To a thoroughly degassed solution of TPGS-750-M (1 mL, 5 wt. % in H_2O) was added NaOH (4 mg, 100 µmol), cBRIDP (2.1 mg, 6 µmol, 0.12 eq.) and palladium allyl chloride (1.8 mg, 5 µmol, 0.1 eq.). The solution was bubbled with nitrogen for 10 minutes followed by addition of the appropriate aryl bromide (0.08 mmol) and appropriate biotransformation mixture (1mL) containing amine **(*S*)-9** (ca. 50 µmol). Reactions were stirred at 50 °C for 24 hours and subsequently quenched by extraction with EtOAc (5 mL). The organics were dried over anhydrous $MgSO_4$. Conversion to and enantiomeric excess of the *N*-arylamine was determined as detailed in the section "Analytics".

Procedure for hydrogen-borrowing amination of 11 followed by *N*-arylation to give 6c

The hydrogen borrowing amination of **11** was performed as previously described.[1] Ammonium chloride buffer (final conc. 1M, pH 9.0, 10 mL), containing NAD (1 mM), purified ChiAmDH (2 mg/mL) and TeSADH G198D/W110A (1 mg/mL) and DMSO (5 % v/v). The substrate **11** (50 mg) was added directly and the reactions were shaken in a bench-top shaker at 30 °C. After 48h, small aliquots of the reactions were quenched by the addition of 10M NaOH (100 µL), and extracted with EtOAc (2 x 350 µL). The combined organics were dried over anhydrous $MgSO_4$. Conversion to amine and enantiomeric excess was analysed by GC-FID as detailed in the section "Analytics". The remainder of the reaction buffer was diluted 1:1 with a thoroughly degassed solution of TPGS-750-M (10 mL, 5 wt. % in H_2O) was added NaOH (40 mg, 1 mmol), cBRIDP (21 mg, 60 µmol, 0.12 eq.) and palladium allyl chloride (18 mg, 50 µmol, 0.1 eq.). The solution was bubbled with nitrogen for 10 minutes followed by addition of **2c** (0.8 mmol) Reactions were stirred at 50 °C for 24 hours and subsequently quenched by extraction with EtOAc (3 x 5 mL). The organics were concentrated under reduced pressure to afford the crude product. The residues were purified by flash chromatography (5–25 % v/v EtOAc/cyclohexane) to afford **6c** (36 mg, 33% yield > 99% *ee* (*R*)) as a yellow oil. ^{1}H NMR ($CDCl_3$, 400 MHz) δ 7.82 (d, *J* = 8.8 Hz, 2 H), 6.59 (d, *J* = 7.1 Hz, 2 H), 3.61 (q, *J* = 6.6 Hz, 1 H), 2.49 (s, 3 H), 1.73 (dt, *J* = 13.5, 6.7 Hz, 1 H), 1.46-1.52 (m, 2 H), 1.34 (dd, *J* = 13.7, 6.9 Hz, 2 H), 1.20 (d, *J* = 6.3 Hz, 3 H) 0.92 (dd, *J* = 13.2, 6.6 Hz, 6 H).

Procedure for the preparative scale reduction of 8 followed by *N*-arylation to give 10a

The reduction of imine **8** (50 mM) was performed using potassium phosphate buffer (final conc. 100 mM, pH 8.0), containing NADP (1 mM), purified S-IRED (1 mg/mL) and DMSO (5 % v/v), and the reaction was shaken in a bench-top shaker at 30 °C (total volume 24 mL). After 48 h, the reaction buffer was added to a solution of toluene (20 mL) containing palladium allyl chloride (44 mg, 0.18 mmol, 10 mol%), PhBr (201 µL, 1.92 mmol, 1.6 eq.), *t*BuXPhos (82 mg, 0.19 mmol, 16 mol%) and KO*t*Bu (270 mg, 2.40 mmol, 1.6 eq.). The solution was stirred vigorously and degassed thoroughly for 30 minutes, after which it was heated to 60 °C for 24 h and subsequently quenched by extraction with EtOAc (3 x 20 mL). The combined organic extracts were washed with saturated brine solution (20 mL) and dried over $MgSO_4$, then were concentrated *in vacuo*. Purification by flash chromatography on silica gel (20 % v/v DCM/hexane) afforded the title compound **10a** (119 mg, 0.74 mmol, 65%, >99% *ee*) as a yellow oil. ^{1}H NMR ($CDCl_3$, 400 MHz) δ 7.24 (td, *J* = 7.9, 7.2, 1.7, 2 H), 6.72-6.51 (m, 3 H), 3.98-3.82 (m, 1 H), 3.44 (dd, *J* = 9.6, 7.5, 1 H), 3.28-3.09 (m, 1 H), 2.18-1.93 (m, 3 H), 1.79-1.67 (m, 1 H), 1.20 (d, *J* = 6.3, 3 H); ^{13}C NMR ($CDCl_3$, 100 MHz) δ 147.4, 129.3, 115.2, 111.9, 53.7, 48.3, 33.2, 23.4, 19.5; HRMS (ESI): Calculated 162.1277 $[M+H]^+$ found 162.1283.

General procedure for the preparation of *N*-arylated amine standards

To a thoroughly degassed solution of TPGS-750-M (1 mL, 2 wt. % in H_2O) was added NaOtBu (115 mg, 1.2 mmol), *t*BuXPhos (51 mg, 0.12 mmol) and palladium allyl chloride (36 mg, 0.1 mmol). The solution was bubbled with nitrogen for 10 minutes followed by addition of the appropriate aryl bromide (1.2 mmol) and amine (1 mmol). Reactions were stirred at 50 °C for 20 hours and subsequently quenched by extraction with EtOAc (3 x 3 mL). The combined organics were concentrated under reduced pressure to afford the crude product. The residues were purified by flash chromatography (5–25 % v/v EtOAc/cyclohexane).

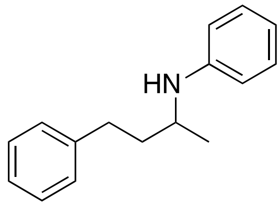

N-(4-phenylbutan-2-yl)aniline (3a): 4-phenyl-2-butanamine **1** (135 mg, 1 mmol) with bromobenzene **2a** (188 mg, 1.2 mmol), afforded **3a** (185 mg, 80 %) as a yellow oil.

^{1}H NMR ($CDCl_3$, 400 MHz) δ , 7.27-7.31 (m, 2 H), 7.13-7.22 (m, 5 H), 6.65-6.69 (m, 1 H), 6.54 (dd, J = 8.5, 0.9 Hz, 2 H), 3.49 (dt, J = 12.7, 6.3 Hz, 1 H), 3.43 (s, 1 H), 2.74 (t, J = 7.9 Hz, 2 H), 1.85-1.94 (m, 1 H), 1.78 (dtd, J = 13.9, 7.9, 6.1 Hz, 1 H) 1.23 (d, J = 6.3 Hz, 3 H).13-C NMR (101 MHz; $CDCl_3$): δ 147.7, 142.1, 129.4, 128.57, 128.52, 126.0, 117.1, 113.3, 48.0, 39.0, 32.6, 21.0. m/z (ES$^-$)
224 (100 %, [M-H]$^-$); (HRMS Found 226.1590, (M+H)$^+$ requires 226.1595)

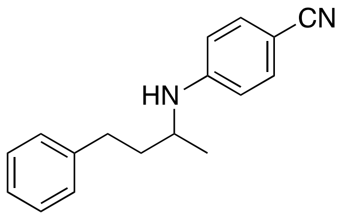

4-((4-phenylbutan-2-yl)amino)benzonitrile (3b): 4-phenyl-2-butanamine **1** (135 mg, 1 mmol) with 4-bromobenzonitrile **2b** (220 mg, 1.2 mmol), **3b** (140 mg, 55 %) as a yellow oil.

^{1}H NMR ($CDCl_3$, 400 MHz) δ , 7.36-7.40 (m, 2 H), 7.26-7.31 (m, 2 H), 7.19-7.23 (m, 1 H), 7.15-7.17 (m, 2 H), 6.42-6.46 (m, 2 H), 4.00 (s, 1 H), 3.51 (m, 1 H), 2.69-2.73 (m, 2 H), 1.79-1.91 (m, 2 H) 1.24 (d, J = 6.4 Hz, 3 H). 13-C NMR (101 MHz; $CDCl_3$): δ 150.7, 141.4, 133.9, 128.65, 128.51, 126.3, 112.5, 98.4, 47.6, 38.5, 32.4, 27.1, 20.7. m/z (ES$^-$) 249 (100 %, [M-H]$^-$). (HRMS Found 273.1360, (M+Na)$^+$ requires 273.1367)

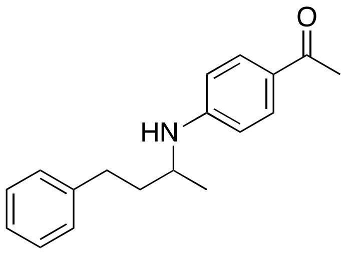

1-(4-((4-phenylbutan-2-yl)amino)phenyl)ethan-1-one (3c): 4-phenyl-2-butanamine **1** (135 mg, 1 mmol) with 4-bromoacetophenone **2c** (240 mg, 1.2 mmol), afforded **3c** (220 mg, 82 %) as a yellow oil.
^{1}H NMR ($CDCl_3$, 400 MHz) δ , 7.78-7.81 (m, 2 H), 7.27-7.31 (m, 2 H), 7.16-7.22 (m, 3 H), 6.44-6.48 (m, 2 H), 4.03 (s, 1 H), 3.57 (m, 1 H), 2.72 (t, *J* = 7.7 Hz, 2 H), 2.49 (s, 3 H), 1.79-1.92 (m, 2 H) 1.25 (d, *J* = 6.3 Hz, 3 H).13-C NMR (101 MHz; $CDCl_3$): δ 151.6, 141.6, 131.0, 128.63, 128.54, 126.2, 111.7, 47.7, 38.7, 32.5, 27.1, 20.9. m/z (ES^+) 306 (100 %, $[M+K]^+$), 290 (95 %, $[M+Na]^+$). (HRMS Found 306.1250, $(M+K)^+$ requires 306.1260)

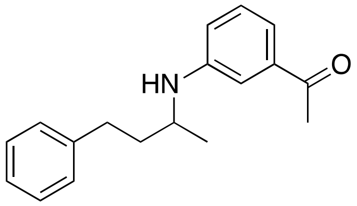

1-(3-((4-phenylbutan-2-yl)amino)phenyl)ethan-1-one (3d): 4-phenyl-2-butanamine **1** (135 mg, 1 mmol) with 3-bromoacetophenone **2d** (220 mg, 1.2 mmol), afforded **3d** (208 mg, 78 %) as a yellow oil.
^{1}H NMR ($CDCl_3$, 400 MHz) δ , 7.12-7.30 (m, 9 H), 6.70 (dt, *J* = 6.5, 2.5 Hz, 1 H), 3.56 (m, 1 H), 2.73 (t, *J* = 7.8 Hz, 2 H), 2.55 (s, 3 H), 1.77-1.91 (m, 2 H) 1.23 (d, *J* = 6.2 Hz, 3 H). 13-C NMR (101 MHz; $CDCl_3$): δ 198.8, 147.9, 141.9, 138.3, 129.5, 128.57, 128.53, 126.1, 117.9, 117.5, 112.1, 48.0, 38.8, 32.6, 26.9, 20.9. m/z (ES^+) 290.2 (100 %, $[M+Na]^+$), 306.2 (50 %, $[M+K]^+$). (HRMS Found 306.1255, $(M+K)^+$ requires 306.1260)

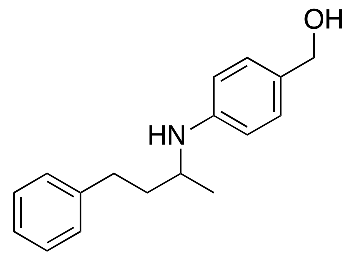

(4-((4-phenylbutan-2-yl)amino)phenyl)methanol (3e): 4-phenyl-2-butanamine **1** (135 mg, 1 mmol) with 4-bromobenzyl alcohol **2e** (224 mg, 1.2 mmol), afforded **3e** (110 mg, 43 %) as a yellow oil.

^{1}H NMR ($CDCl_3$, 400 MHz) δ , 7.32-7.37 (m, 2 H), 7.20-7.26 (m, 5 H), 6.62 (d, *J* = 8.4 Hz, 2 H), 4.60 (s, 2 H), 3.55 (q, *J* = 6.3 Hz, 1 H), 2.79 (t, *J* = 7.5 Hz, 2 H), 1.84-1.97 (m, 2 H) 1.29 (d, *J* = 6.3 Hz, 3 H). 13-C NMR (101 MHz; $CDCl_3$): δ 141.9, 131.7, 129.0, 128.70, 128.54, 128.52, 126.0, 113.6, 65.5, 48.5, 38.7, 32.5, 20.7. m/z (ES$^+$) 278.1 (100 %, [M+Na]$^+$). (HRMS Found 294.1251, (M+K)$^+$ requires 294.1260)

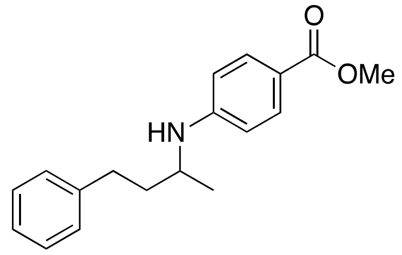

methyl 4-((4-phenylbutan-2-yl)amino)benzoate (3f): 4-phenyl-2-butanamine **1** (135 mg, 1 mmol) with 4-bromomethyl benzoate **2f** (260 mg, 1.2 mmol), afforded **3f** (165 mg, 58 %) as a yellow oil.

^{1}H NMR ($CDCl_3$, 400 MHz) δ , 7.81-7.84 (m, 2 H), 7.26-7.30 (m, 2 H), 7.16-7.22 (m, 3 H), 6.43-6.47 (m, 2 H), 3.94 (s, 1 H), 3.84 (s, 3 H), 3.55 (m, 1 H), 2.72 (t, *J* = 7.7 Hz, 2 H), 1.78-1.91 (m, 2 H) 1.24 (d, *J* = 6.3 Hz, 3 H). 13-C NMR (101 MHz; $CDCl_3$): δ 167.5, 151.4, 141.7, 131.7, 128.61, 128.53, 126.2, 118.0, 111.8, 51.6, 47.7, 38.7, 32.5, 20.8. m/z (ES$^+$) 306.2 (100 %, [M+Na]$^+$), 322.1 (40 %, [M+K]$^+$). (HRMS Found 306.1462, (M+Na)+ requires 306.1469)

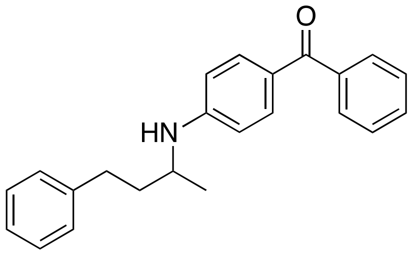

phenyl(4-((4-phenylbutan-2-yl)amino)phenyl)methanone (3g): 4-phenyl-2-butanamine **1** (135 mg, 1 mmol) with 4-bromobenzophenone **2g** (315 mg, 1.2 mmol), afforded **3g** (232 mg, 70 %) as a yellow oil.
^{1}H NMR ($CDCl_3$, 400 MHz) δ , 7.68-7.74 (m, 4 H), 7.43-7.55 (m, 3 H), 7.27-7.31 (m, 2 H), 7.16-7.24 (m, 3 H), 6.49 (q, *J* = 4.8 Hz, 2 H), 4.05 (d, *J* = 8.2 Hz, 1 H), 3.54-3.62 (m, 1 H), 2.73 (t, *J* = 7.7 Hz, 2 H) 1.79-1.96 (m, 2 H). 13-C NMR (101 MHz; $CDCl_3$): δ 195.2, 151.5, 141.6, 139.4, 133.2, 131.3, 129.6, 128.64, 128.53, 128.2, 126.2, 125.9, 111.7, 47.7, 38.7, 32.5, 20.9. m/z (ES^+) 368.2 (100 %, $[M+K]^+$), 352.2 (70 %, $[M+Na]^+$). (HRMS Found 352.1670, (M+Na)+ requires 352.1677)

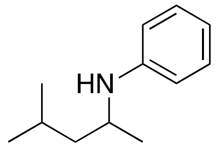

***N*-(4-methylpentan-2-yl)aniline (6a):** dimethylbutyl amine **5** (100 mg, 0.98 mmol) with bromobenzene **2a** (142 mg, 1.2 mmol), afforded **6a** (165 mg, 73 %) as a yellow oil.
H NMR ($CDCl_3$, 400 MHz) δ , 7.15-7.20 (m, 2 H), 6.68 (t, *J* = 7.3 Hz, 1 H), 6.60 (dd, *J* = 8.5, 0.9 Hz, 2 H), 3.55 (m, 1 H), 3.38 (s, 1 H), 1.77 (dt, *J* = 13.5, 6.8 Hz, 1 H), 1.49 (dt, *J* = 13.7, 6.9 Hz, 1 H), 1.29 (dd, *J* = 13.6, 6.9 Hz, 1 H), 1.18 (d, *J* = 6.2 Hz, 3 H) 0.95 (dd, *J* = 14.1, 6.6 Hz, 6 H). 13-C NMR (101 MHz; $CDCl_3$): δ 147.8, 129.4, 116.8, 113.1, 47.1, 46.6, 25.2, 23.1, 22.7, 21.2. m/z (EI) 120.1 (100 %, $[M-C_4H_9]^{+\cdot}$), 177.1 (20 %, $[M]^{+\cdot}$). (HRMS Found 178.1588, (M+H)+ requires 178.1595)

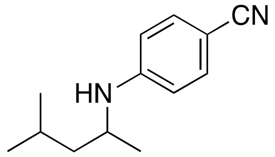

4-((4-methylpentan-2-yl)amino)benzonitrile (6b): dimethylbutyl amine **5** (100 mg, 0.98 mmol) with 4-bromobenzonitrile **2b** (218 mg, 1.2 mmol), afforded **6b** (143 mg, 57 %) as a yellow oil.
^{1}H NMR ($CDCl_3$, 400 MHz) δ , 7.91 (dd, *J* = 9.2, 2.2 Hz, 2 H), 6.53 (dd, *J* = 9.2, 2.1 Hz, 2 H), 3.62 (m, 1 H), 1.73 (dt, *J* = 13.5, 6.7 Hz, 1 H), 1.48 (dt, *J* = 13.8, 7.0 Hz, 1 H), 1.28-1.35 (m, 1 H), 1.19 (d, *J* = 6.3 Hz, 3 H) 0.93 (dd, *J* = 16.0, 6.6 Hz, 6 H). 13-C NMR (101 MHz; $CDCl_3$): δ 150.8, 133.9, 133.5, 112.4, 98.1, 46.6, 46.4, 25.2, 22.9, 22.7, 20.9. m/z (EI) 145.1 (100 %, $[M-C_4H_9]^{+\cdot}$), 202.1 (20 %, $[M]^{+\cdot}$). (HRMS Found 203.1536, (M+H)+ requires 203.1548)

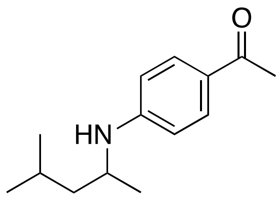

1-(4-((4-methylpentan-2-yl)amino)phenyl)ethan-1-one (6c): dimethylbutyl amine **5** (100 mg, 0.98 mmol) with 4-bromoacetophenone **2c** (238 mg, 1.2 mmol), afforded **6c** (215 mg, 80 %) as a yellow oil.

^{1}H NMR ($CDCl_3$, 400 MHz) δ , 7.79-7.83 (m, 2 H), 6.50-6.54 (m, 2 H), 4.00 (d, *J* = 0.5 Hz, 1 H), 3.62 (m, 1 H), 2.48 (s, 3H), 1.68-1.76 (m, 1 H), 1.47 (dt, *J* = 13.8, 7.0 Hz, 1 H), 1.31 (dt, *J* = 13.8, 6.9 Hz, 1 H), 1.19 (d, *J* = 6.3 Hz, 3 H) 0.93 (dd, *J* = 16.5, 6.6 Hz, 6 H). 13-C NMR (101 MHz; $CDCl_3$): δ 196.3, 151.7, 131.1, 126.3, 111.6, 46.7, 46.4, 26.1, 25.2, 22.9, 22.7, 21.1. m/z (EI) 162.1 (100 %, $[M-C_4H_9]^{+\cdot}$), 219.1 (20 %, $[M]^{+\cdot}$). (HRMS Found 220.1688, (M+H)+ requires 220.1701)

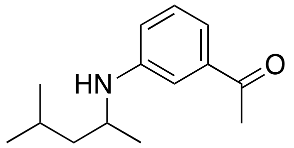

1-(3-((4-methylpentan-2-yl)amino)phenyl)ethan-1-one (6d): dimethylbutyl amine **5** (100 mg, 0.98 mmol) with 3-bromoacetophenone **2d** (238 mg, 1.2 mmol), afforded **6d** (197 mg, 74 %) as a yellow oil.

^{1}H NMR ($CDCl_3$, 400 MHz) δ , 7.21-7.23 (m, 2 H), 7.15 (t, *J* = 1.4 Hz, 1 H), 6.73-6.78 (m, 1 H), 3.59 (m, 1 H), 2.57 (s, 3 H), 1.74 (dq, *J* = 13.5, 6.7 Hz, 1 H), 1.47 (dt, *J* = 13.6, 6.8 Hz, 1 H), 1.28 (dt, *J* = 13.5, 6.7 Hz, 1 H), 1.16 (d, *J* = 5.8 Hz, 3 H) 0.93 (dd, *J* = 17.6, 6.6 Hz, 6 H). 13-C NMR (101 MHz; $CDCl_3$): δ 198.8, 148.0, 138.4, 129.5, 117.8, 117.3, 111.9, 47.0, 46.6, 26.9, 25.2, 23.0, 22.8, 21.1. m/z (EI) 162.1 (100 %, $[M-C_4H_9]^{+\cdot}$), 219.1 (25 %, $[M]^{+\cdot}$). (HRMS Found 220.1692, (M+H)+ requires 220.1701)

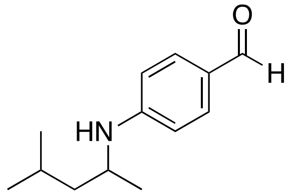

4-((4-methylpentan-2-yl)amino)benzaldehyde (6e): dimethylbutyl amine **5** (100 mg, 0.98 mmol) with 4-bromobenzyl alcohol **2e** (222 mg, 1.2 mmol), afforded **6e** (136 mg, 54 %) as a yellow oil.

^{1}H NMR ($CDCl_3$, 400 MHz) δ , 9.70 (s, 1 H), 7.66-7.69 (m, 2 H), 6.57 (d, *J* = 8.7 Hz, 2 H), 4.14 (s, 1 H), 3.64 (m, 1 H), 1.73 (dt, *J* = 13.5, 6.8 Hz, 1 H), 1.48 (dt, *J* = 13.9, 7.0 Hz, 1 H), 1.33 (dd, *J* = 13.8, 6.9 Hz, 1 H), 1.20 (d, *J* = 6.3 Hz, 3 H) 0.92 (dt, *J* = 15.1, 6.9 Hz, 6 H). 13-C NMR (101 MHz; $CDCl_3$): δ 190.2, 152.9, 132.58, 126.2, 112.0, 46.7, 46.5, 25.2, 22.9, 22.7, 21.0. m/z (EI) 148.1 (100 %, $[M-C_4H_9]^{+\cdot}$), 205.1 (20 %, $[M]^{+\cdot}$). (HRMS Found 206.1534, (M+H)+ requires 206.1544)

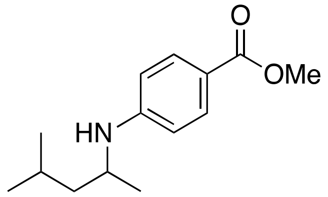

Methyl 4-((4-methylpentan-2-yl)amino)benzoate (6f): dimethylbutyl amine **5** (100 mg, 0.98 mmol) with 4-bromomethyl benzoate **2f** (258 mg, 1.2 mmol), afforded **6f** (169 mg, 60 %) as a yellow oil
^{1}H NMR ($CDCl_3$, 400 MHz) δ , 7.82-7.85 (m, 2 H), 6.50-6.52 (m, 2 H), 3.89 (m, 1 H), 3.84 (s, 3 H), 1.73 (t, *J* = 6.7 Hz, 1 H), 1.45 (dt, *J* = 13.0, 5.9 Hz, 1 H), 1.29 (dt, *J* = 13.7, 6.9 Hz, 2 H), 1.17 (d, *J* = 6.3 Hz, 3 H) 0.92 (dd, *J* = 15.9, 6.6 Hz, 6 H). 13-C NMR (101 MHz; $CDCl_3$): δ 167.5, 151.5, 131.8, 117.8, 111.6, 51.6, 46.8, 46.4, 25.2, 23.0, 22.7, 21.0. m/z (EI) 164.1 (100 %, $[M-C_4H_9]^{+\cdot}$), 221.1 (10 %, $[M-CH_3+H]^{+\cdot}$). (HRMS Found 221.1482, $(M-CH_3+H)+$ requires 222.1494)

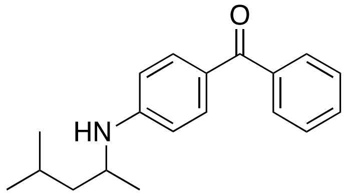

(4-((4-methylpentan-2-yl)amino)phenyl)(phenyl)methanone (6g): dimethylbutyl amine **5** (100 mg, 0.98 mmol) with 4-bromobenzophenone **2g** (313 mg, 1.2 mmol), afforded **6g** (176 mg, 53 %) as a yellow oil.
^{1}H NMR ($CDCl_3$, 400 MHz) δ , 7.26-7.30 (m, 3 H), 6.98-7.10 (m, 4 H), 6.09-6.12 (m, 2 H), 3.63 (s, 1 H), 3.19 (m, 1 H), 1.29 (dd, *J* = 13.4, 6.7 Hz, 1 H), 1.04 (dt, *J* = 13.8, 7.0 Hz, 1 H), 0.87 (dt, *J* = 13.8, 6.9 Hz, 1 H), 0.75 (d, *J* = 6.3 Hz, 3 H) 0.49 (dd, *J* = 14.4, 6.6 Hz, 6 H). 13-C NMR (101 MHz; $CDCl_3$): δ 195.2, 151.8, 139.5, 133.4, 131.3, 129.6, 125.7, 111.6, 46.8, 46.5, 25.3, 23.0, 22.8, 21.2. m/z (EI) 224.1 (100 %, $[M-C_4H_9]^{+\cdot}$), 281.2 (20 %, $[M]^{+\cdot}$). (HRMS Found 282.1843, (M+H)+ requires 282.1857)

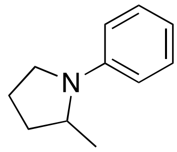

2-methyl-1-phenylpyrrolidine (10a): 2-methylpyrolidine **9** (85 mg, 1 mmol) with 4-bromobenzene **2a** (188 mg, 1.2 mmol), afforded **10a** (110 mg, 68 %) as a yellow oil.
^{1}H NMR (CDCl3, 400 MHz) δ, 7.21-7.26 (m, 2 H), 6.65 (t, J = 7.3 Hz, 1 H), 6.59 (d, J = 8.0 Hz, 2 H), 3.87-3.90 (m, 1 H), 3.43 (td, J = 8.4, 2.0 Hz, 1 H), 3.13-3.18 (m, 1 H), 1.96-2.10 (m, 3 H), 1.70-1.72 (m, 1 H) 1.18 (d, J = 6.2 Hz, 3 H). ^{13}C NMR (101 MHz; CDCl3): δ 147.4, 129.3, 115.2, 111.9, 53.7, 48.3, 33.3, 23.4, 19.5. m/z (EI) 146.1 (100 %, $[M-CH_3]^{+\cdot}$), 177.1 (40 %, $[M]^{+\cdot}$). (HRMS Found 162.1281, (M+H)+ requires 162.1282)

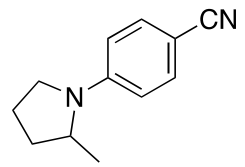

4-(2-methylpyrrolidin-1-yl)benzonitrile (10b): 2-methylpyrolidine **9** (85 mg, 1 mmol) with 4-bromobenzonitrile **2b** (218 mg, 1.2 mmol), afforded **10b** (98 mg, 52 %) as a yellow oil.
^{1}H NMR (CDCl3, 400 MHz) δ , 7.45 (d, J = 8.9 Hz, 2 H), 6.56 (d, J = 8.6 Hz, 2 H), 3.94 (quintet, J = 6.0 Hz, 1 H), 3.45 (dd, J = 9.6, 7.7 Hz, 1 H), 3.18-3.25 (m, 1 H), 2.01-2.16 (m, 3 H) 1.73-1.82 (m, 1 H) 1.18 (d, J = 6.3 Hz, 3 H). ^{13}C NMR (101 MHz; CDCl3): δ 149.0, 120.8, 120.2, 112.70, 112.51, 55.03, 48.76, 32.9, 23.1, 18.5 m/z (EI) 171.1 (100 %, [M-CH3]+·), 186.1 (20%, [M]+·). (HRMS Found 187.228, (M+H)+ requires 187.1235)

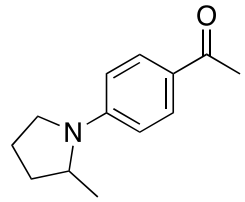

1-(4-(2-methylpyrrolidin-1-yl)phenyl)ethan-1-one (10c): 2-methylpyrolidine **9** (85 mg, 1 mmol) with 4-bromoacetophenone **2c** (238 mg, 1.2 mmol), afforded **10c** (172 mg, 85 %) as a yellow oil.
^{1}H NMR ($CDCl_3$, 400 MHz) δ , 7.84-7.87 (m, 2 H), 6.52-6.54 (m, 2 H), 3.99 (d, *J* = 6.1 Hz, 1 H), 3.46-3.48 (m, 1 H), 3.24-3.27 (m, 1 H), 2.50 (d, *J* = 5.3 Hz, 3 H), 2.03-2.12 (m, 3 H), 1.74-1.77 (m, 1 H) 1.20 (d, *J* = 6.3 Hz, 3 H). 13-C NMR (101 MHz; $CDCl_3$): δ 196.4, 150.4, 130.8, 124.9, 111.0, 53.9, 48.0, 33.0, 23.2, 19.0. m/z (EI) 188.1 (100 %, $[M-CH_3]^{+\cdot}$), 203.1 (25 %, $[M]^{+\cdot}$). (HRMS Found 226.1200, (M+Na)+ requires 226.1207)

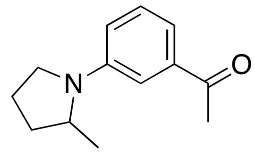

1-(3-(2-methylpyrrolidin-1-yl)phenyl)ethan-1-one (10d): 2-methylpyrolidine **9** (85 mg, 1 mmol) with 3-bromoacetophenone **2d** (238 mg, 1.2 mmol), afforded **5d** (151 mg, 74 %) as a yellow oil.
^{1}H NMR ($CDCl_3$, 400 MHz) δ , 7.28 (t, *J* = 7.9 Hz, 1 H), 7.19-7.21 (m, 1 H), 7.14 (t, *J* = 2.1 Hz, 1 H), 6.75-6.78 (m, 1 H), 3.91-3.94 (m, 1 H), 3.46 (ddd, *J* = 9.4, 7.5, 2.1 Hz, 1 H), 3.17-3.23 (m, 1 H), 2.58 (s, 3 H), 1.98-2.13 (m, 3 H), 1.70-1.76 (m, 1 H) 1.18 (d, *J* = 6.2 Hz, 3 H). 13-C NMR (101 MHz; $CDCl_3$): δ 199.2, 147.3, 138.1, 129.3, 116.5, 115.7, 110.7, 53.8, 48.3, 33.2, 26.9, 23.3, 19.2. m/z (EI) 188.1 (100 %, $[M-C_4H_9]^{+\cdot}$), 203.1 (20 %, $[M]^{+\cdot}$). (HRMS Found 226.1200, (M+Na)+ requires 226.1207)

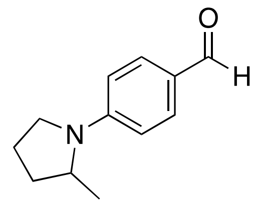

4-(2-methylpyrrolidin-1-yl)benzaldehyde (10e): 2-methylpyrolidine **9** (85 mg, 1 mmol) with 4-bromobeenzyl alcohol **2e** (224 mg, 1.2 mmol), afforded **10e** (101 mg, 53 %) as a yellow oil.

^{1}H NMR ($CDCl_3$, 400 MHz) δ , 9.71 (s, 1 H), 7.70-7.74 (m, 2 H), 6.59 (d, *J* = 8.9 Hz, 2 H), 4.02 (t, *J* = 6.2 Hz, 1 H), 3.50 (td, *J* = 9.1, 1.9 Hz, 1 H), 3.25-3.31 (m, 1 H), 2.02-2.17 (m, 3 H), 1.76-1.79 (m, 1 H) 1.21 (d, *J* = 6.3 Hz, 3 H). 13-C NMR (101 MHz; $CDCl_3$): δ 190.3, 151.4, 132.30, 124.7, 111.5, 54.0, 48.0, 32.9, 23.1, 18.8. m/z (EI) 174.1 (100 %, $[M\text{-}CH_3]^{+\cdot}$), 189.1 (20 %, $[M]^{+\cdot}$). (HRMS Found 214.1200, (M+Na)+ requires 214.1207)

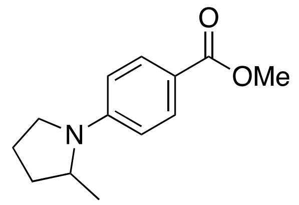

Methyl 4-(2-methylpyrrolidin-1-yl)benzoate (10f): 2-methylpyrolidine **9** (85 mg, 1 mmol) with 4-bromomethyl benzoate **2f** (258 mg, 1.2 mmol), afforded **10f** (117 mg, 53 %) as a yellow oil.

^{1}H NMR ($CDCl_3$, 400 MHz) δ , 7.89-7.93 (m, 2 H), 6.53-6.56 (m, 2 H), 4.00 (dq, *J* = 12.9, 6.8 Hz, 1 H), 3.87 (s, 3 H), 3.46-3.51 (m, 1 H), 3.22-3.29 (m, 1 H), 2.01-2.14 (m, 3 H) 1.75-1.80 (m, 1 H) 1.21 (d, *J* = 6.3 Hz, 3 H). 13-C NMR (101 MHz; $CDCl_3$): δ 167.6, 150.2, 131.4, 116.1, 110.8, 53.7, 51.4, 47.9, 32.7, 23.1, 18.9. m/z (EI) 188.1 (100 %, $[M\text{-}OCH_3]^{+\cdot}$). (HRMS Found 242.0909, (M+Na)+ requires 242.1156)

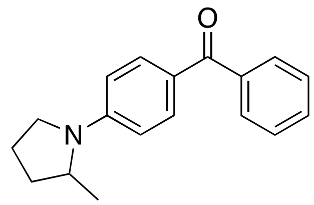

(4-(2-methylpyrrolidin-1-yl)phenyl)(phenyl)methanone (10g): 2-methylpyrolidine **9** (85 mg, 1 mmol) with 4-bromobenzophenone **2g** (313 mg, 1.2 mmol), afforded **10g** (165 mg, 62 %) as a yellow oil.

^{1}H NMR ($CDCl_3$, 400 MHz) δ , 7.77-7.81 (m, 2 H), 7.70-7.73 (m, 2 H), 7.42-7.54 (m, 3 H), 6.55-6.58 (m, 2 H), 3.98-4.05 (m, 1 H), 3.48-3.53 (m, 1 H), 3.24-3.31 (m, 1 H), 2.02-2.17 (m, 3 H), 1.73-1.79 (m, 1 H) 1.21 (q, *J* = 6.7 Hz, 3 H). 13-C NMR (101 MHz; $CDCl_3$): δ 195.1, 150.4, 139.6, 133.1, 131.0, 129.5, 128.1, 124.1, 110.9, 53.9, 48.0, 32.9, 23.1, 18.9. m/z (EI) 250.1 (100 %, $[M\text{-}CH_3]^{+\cdot}$), 265.1 (15 %, $[M]^{+\cdot}$). (HRMS Found 288.1353, (M+Na)+ requires 288.1364)

Analytics

Method for determination of conversion:

a) Column Agilent HP-1 (30 m, 250 µm, 0.25 µm), GC program parameters; injector 250 °C; constant pressure 6.84 psi; temperature program: 50 °C/hold 2 min; 90 °C/10 °C min^{-1}/hold 0 min; 200 °C/ rate 20 °C min^{-1}/hold 1.5 min.

Table 2 Retention times of substrates, intermediates and products

Compound	Retention time (min)
**1**	9.7
**3a**	14.4
**3b**	17.6
**3c**	20.3
**3d**	17.0
**3e**	14.4
**3f**	16.8
**3g**	17.4
**5**	3.9
**6a**	10.8
**6b**	13.7
**6c**	14.2
**6d**	13.4
**6e**	13.7
**6f**	14.6
**6g**	17.6
**9**	3.6
**10a**	11.0
**10b**	14.3
**10c**	14.8
**10d**	13.8
**10e**	20.2
**10f**	13.8
**10g**	18.0

Methods for determination of enantiomeric excess

Enantiomeric excess of amines **1** and **5** was determined by GC-FID as previously reported.[1] Enantiomeric excess of **9** was determined by GC-FID as the *N*-trifluoroacetyl derivatives. Samples used for determination of conversion (200 µL) were diluted with EtOAc (200 µL), treated with trifluorocetic anhydride (10 µL) and Et_3N (5 µL) and shaken at RT for 1 hour. The reaction was quenched by the addition of water (100 µL) and the organics were dried over anhydrous $MgSO_4$. GC program parameters: Varian Chrompack Chiracel Dex-CB (25 m, 320 µm, 0.25 µm) injector 200°C; constant flow 1.7 mL; temperature program: 100 °C/hold 2 min; 130 °C/rate 1 °C min-1/hold 5 min; 170 °C/rate 10 °C min- 1/hold 10 min; 180 °C/rate 10 °C min-1/hold 1 min. Retention time (*R*)-**9** 9.9 min, (*S*)-**9** 10.1 min

Enantiomeric excess of *N*-arylamines was determined by HPLC using the following methods:

a) Column Chiralpak OD-H (250mm ×4.6 mm) column, flow rate 1 mL min^{-1}. Mobile phase Hexane:2-propanol 98:2 + 0.01% diethyl amine.
b) Column Chiralpak OD-H (250mm ×4.6 mm) column, flow rate 1 mL min^{-1}. Mobile phase Hexane:2-propanol 95:5 + 0.01% diethyl amine.
c) Column Chiralpak OD-H (250mm ×4.6 mm) column, flow rate 0.7 mL min^{-1}. Mobile phase Hexane:2-propanol 90:10 + 0.01% diethyl amine.
d) Column Chiralpak OD-H (250mm ×4.6 mm) column, flow rate 0.7 mL min^{-1}. Mobile phase Hexane:2-propanol 80:20 + 0.01% diethyl amine.

Table 3 Retention times

Substrate	Method	Retention time (min)	
		(*R*)-amine	(*S*)-amine
**3a**	b	8.6	9.9
**3b**	d	13.2	21.6
**3c**	d	18.4	21.3
**3d**	d	15.0	17.8
**3e**	d	19.3	20.0
**3f**	d	16.7	17.6
**3g**	*	-	-
**6a**	a	10.2	10.4
**6b**	b	8.1	12.7
**6c**	b	11.1	15.1
**6d**	b	9.2	10.3
**6e**	b	14.8	24.2
**6f**	b	7.9	8.8
**6g**	b	14.3	14.8
**10a**	*	-	-
**10b**	*	-	-
**10c**	a	18.6	19.4
**10d**	d	10.5	22.3
**10e**	b	14.1	15.4
**10f**	d	10.6	22.7
**10g**	a	23.7	24.5

* Could not be separated with any of methods a-d

GC-FID for determination of enantiomeric excess of (*R*)-1

Print of all graphic windows

Current Chromatogram(s)

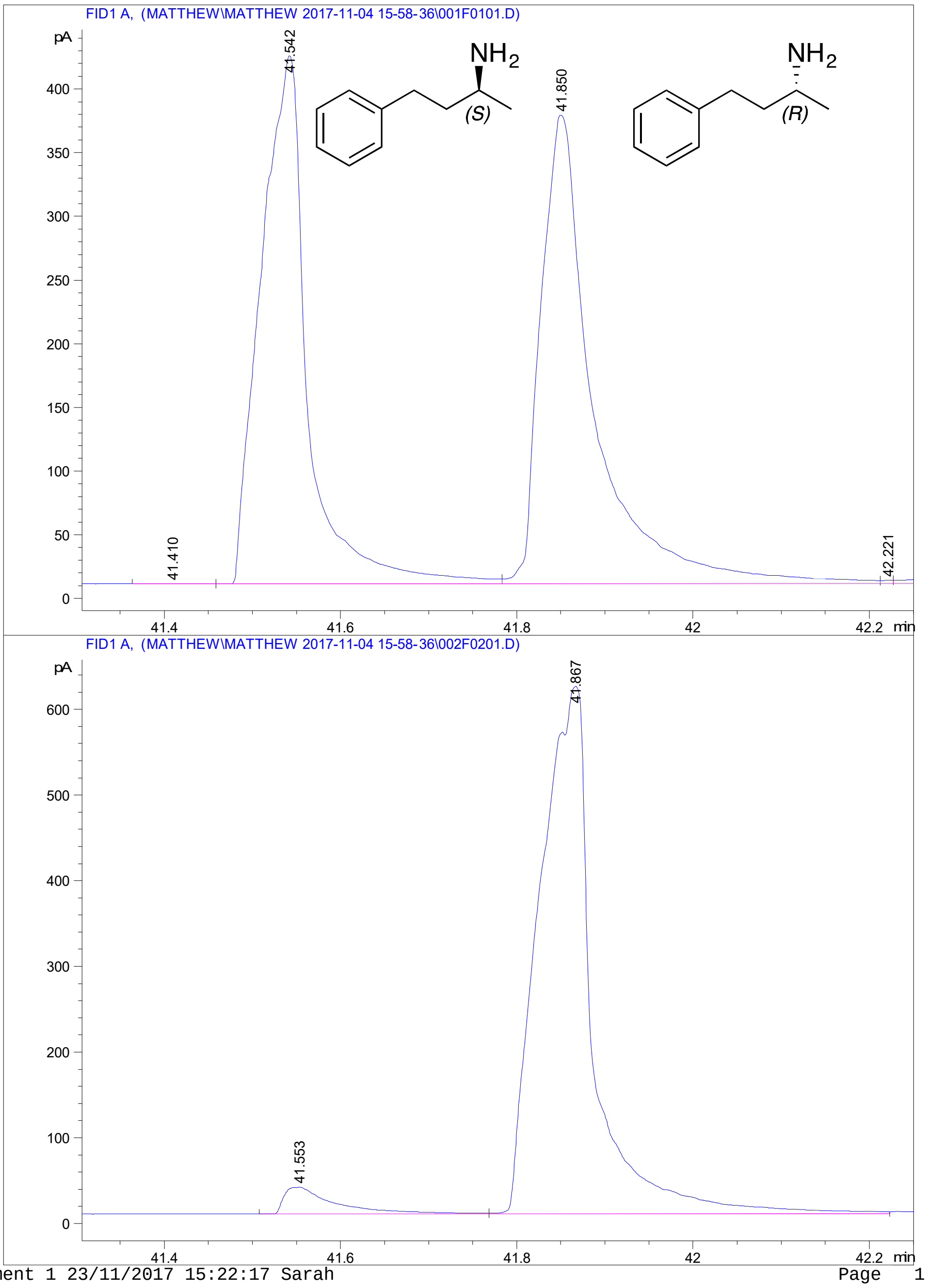

Instrument 1 23/11/2017 15:22:17 Sarah Page 1 of 1

GC-FID for determination of enantiomeric excess of (*R*)-5

Print of all graphic windows

Current Chromatogram(s)

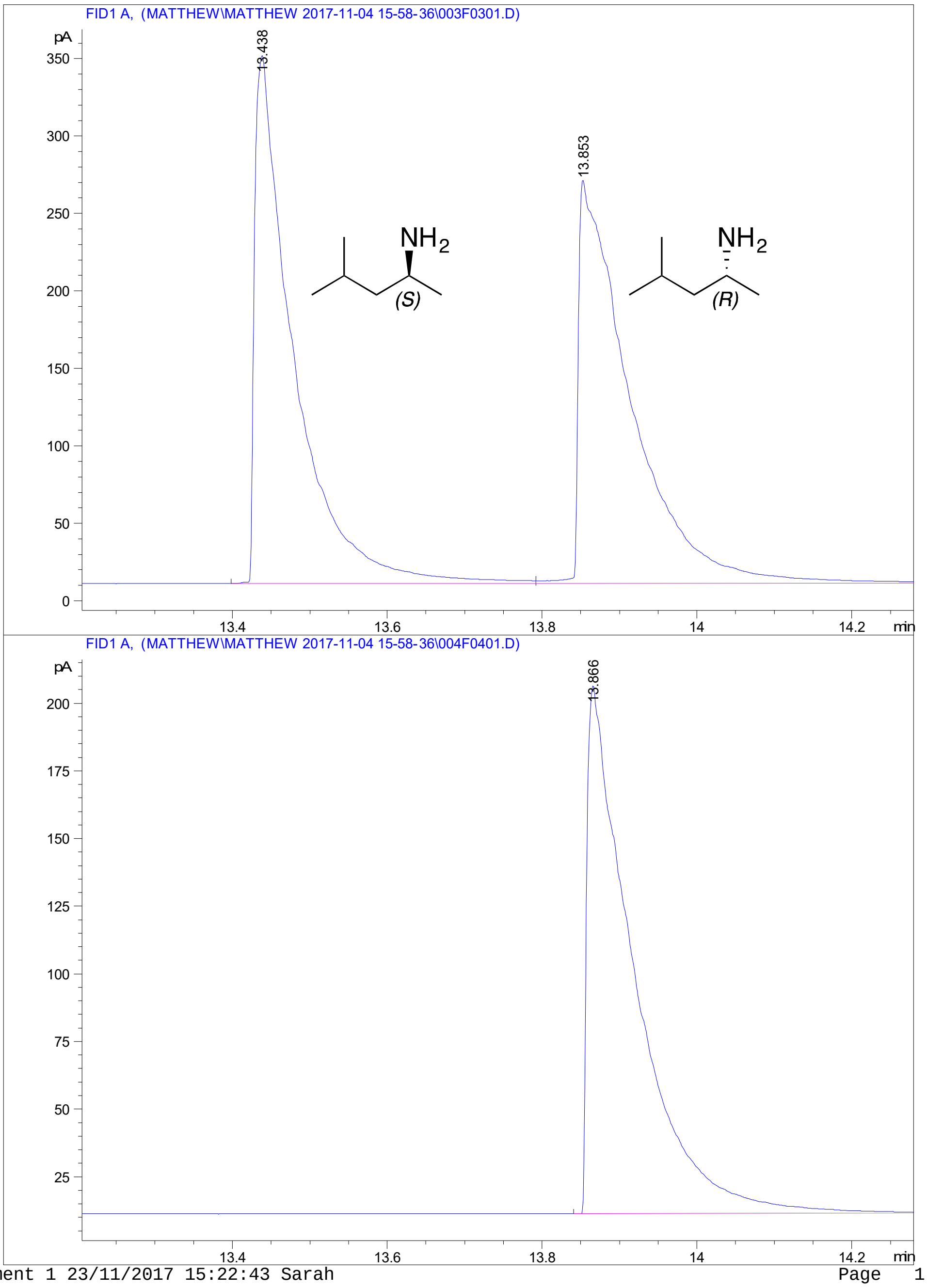

Instrument 1 23/11/2017 15:22:43 Sarah Page 1 of 1

GC-FID for determination of enantiomeric excess of (*S*)-

Print of all graphic windows

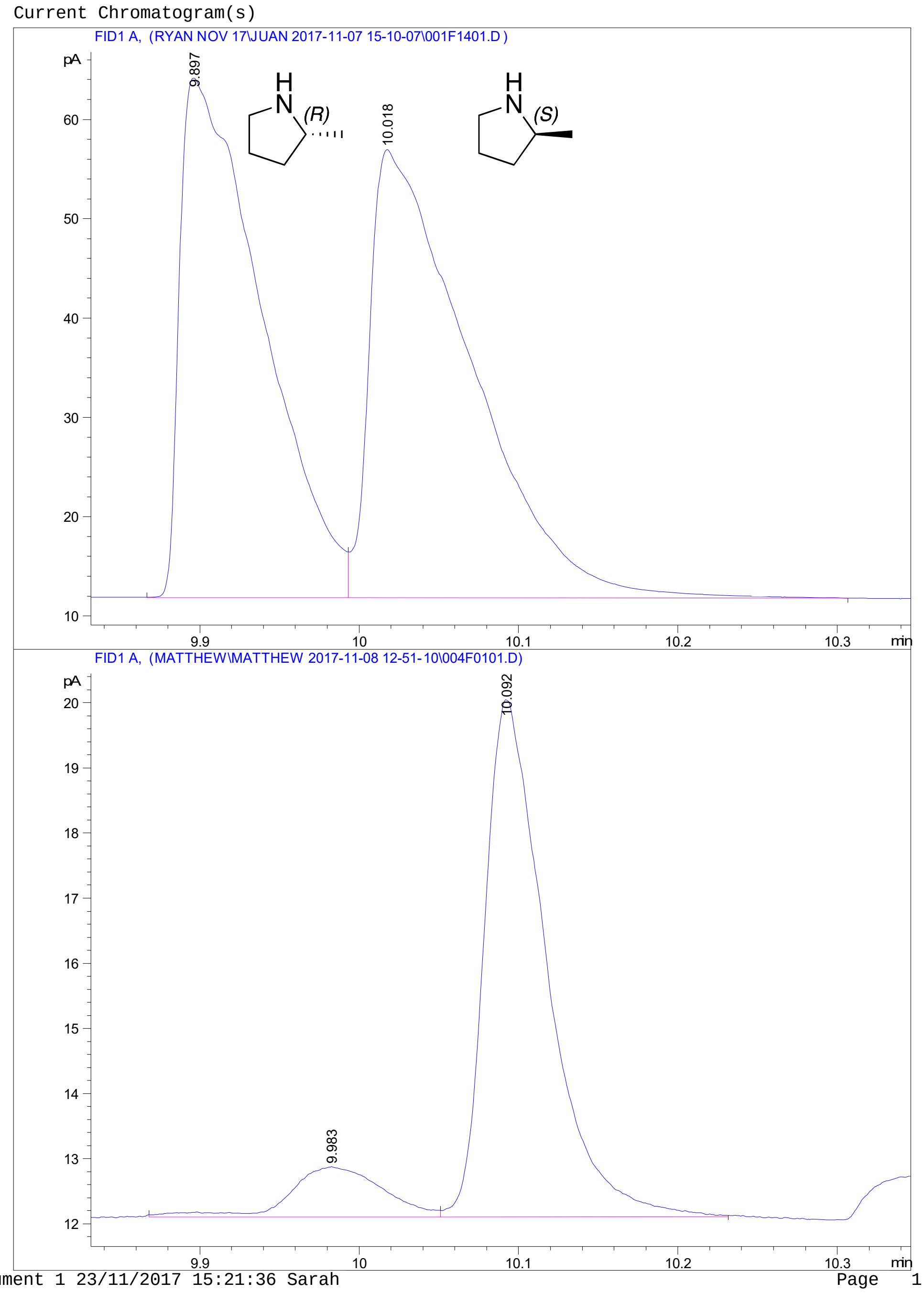

Instrument 1 23/11/2017 15:21:36 Sarah Page 1 of 1

3a - ^{1}H NMR

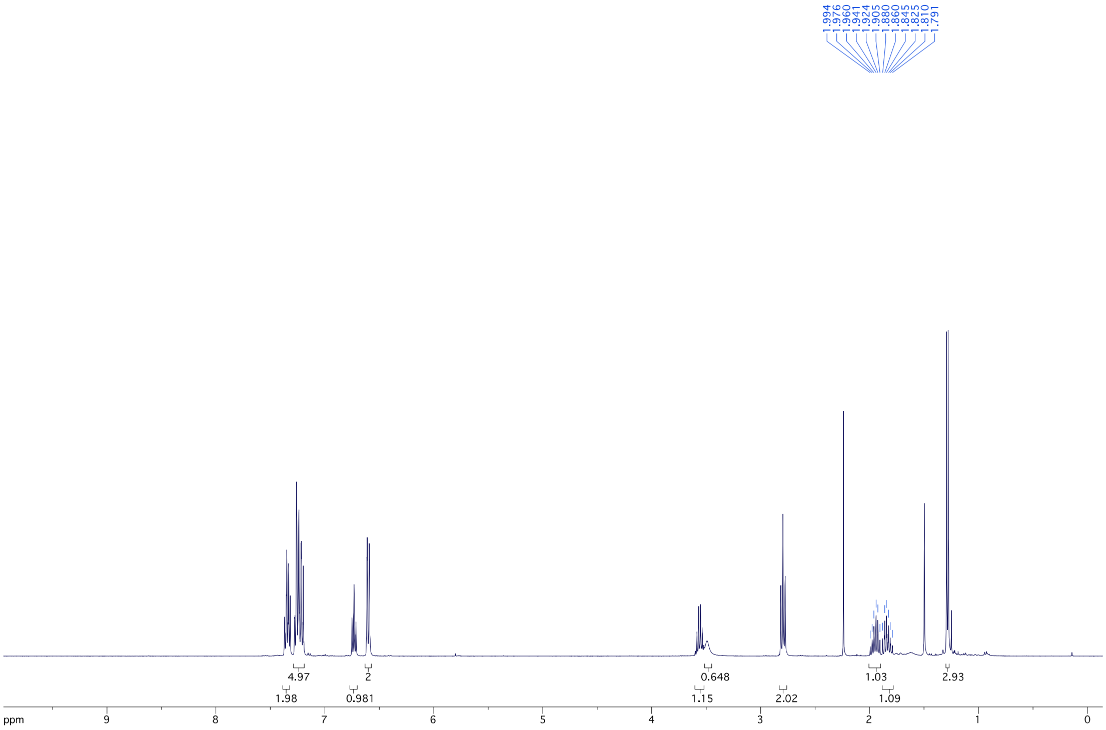

3a – ^{13}C NMR

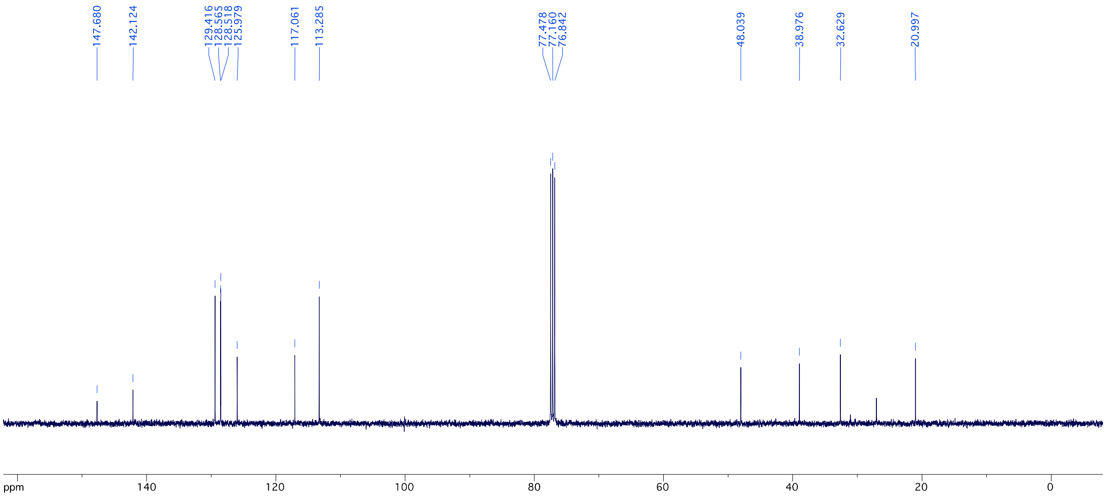

3b – ^{1}H NMR

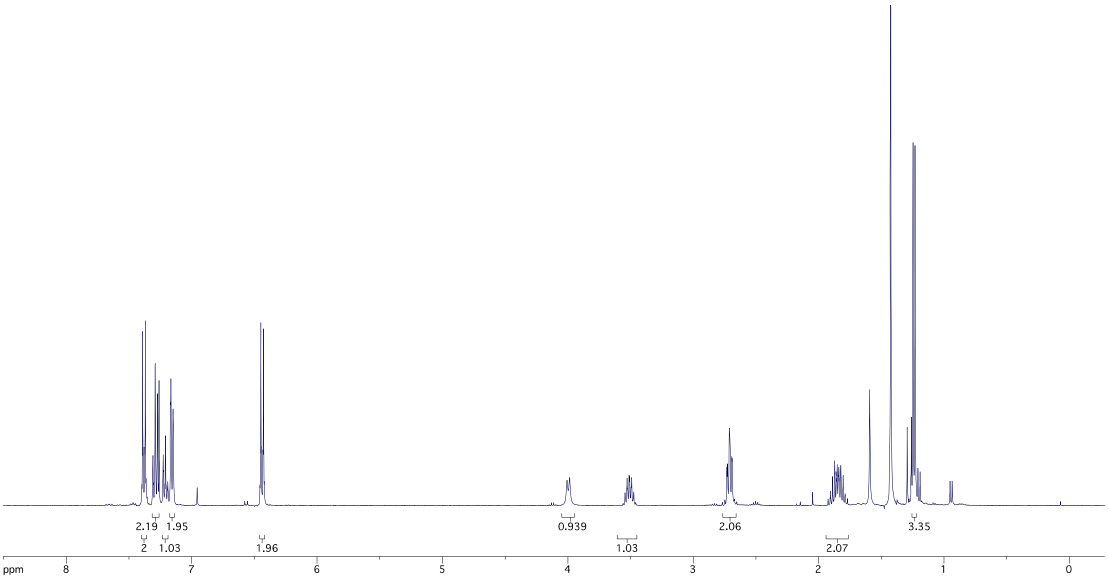

3b – ^{13}C NMR

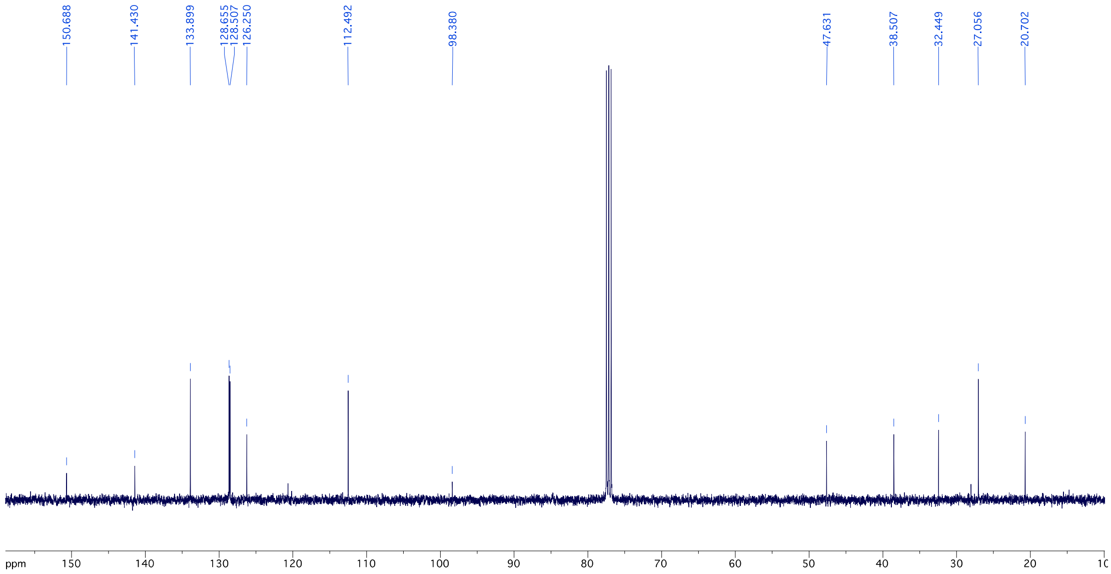

3c – ^{1}H NMR

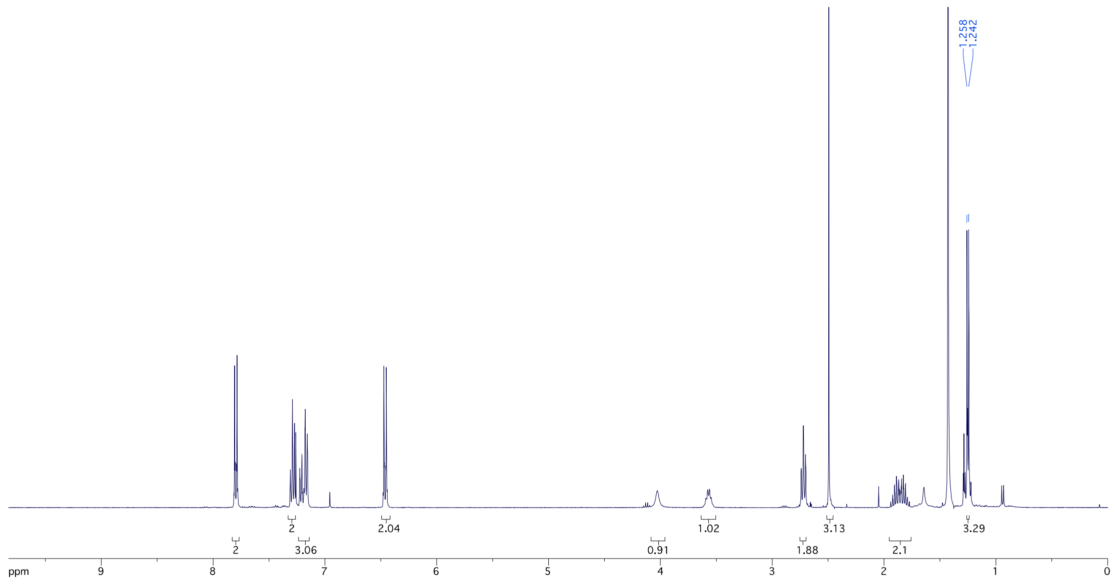

3c – ^{13}C NMR

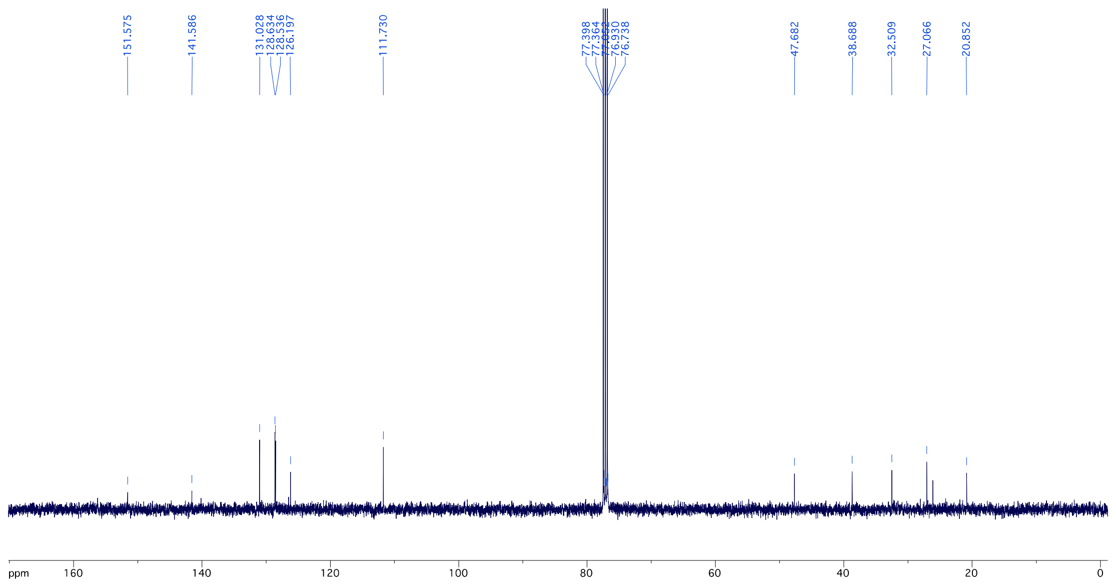

3d – ^{1}H NMR

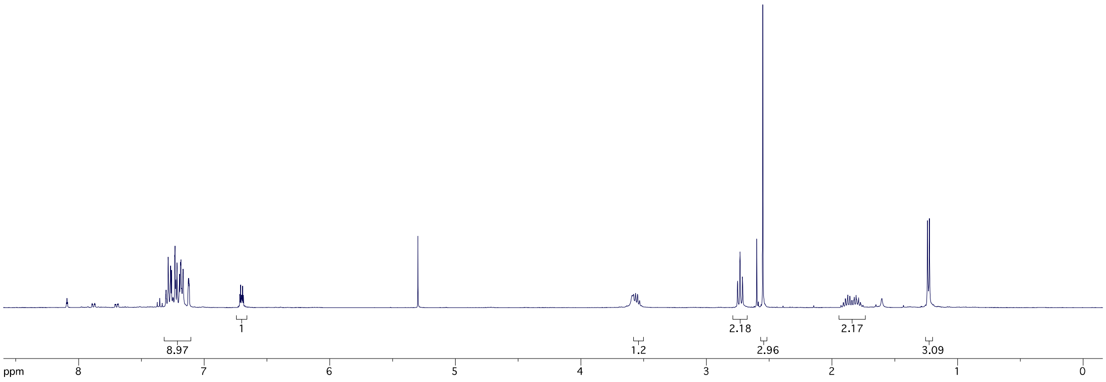

3d – ^{13}C NMR

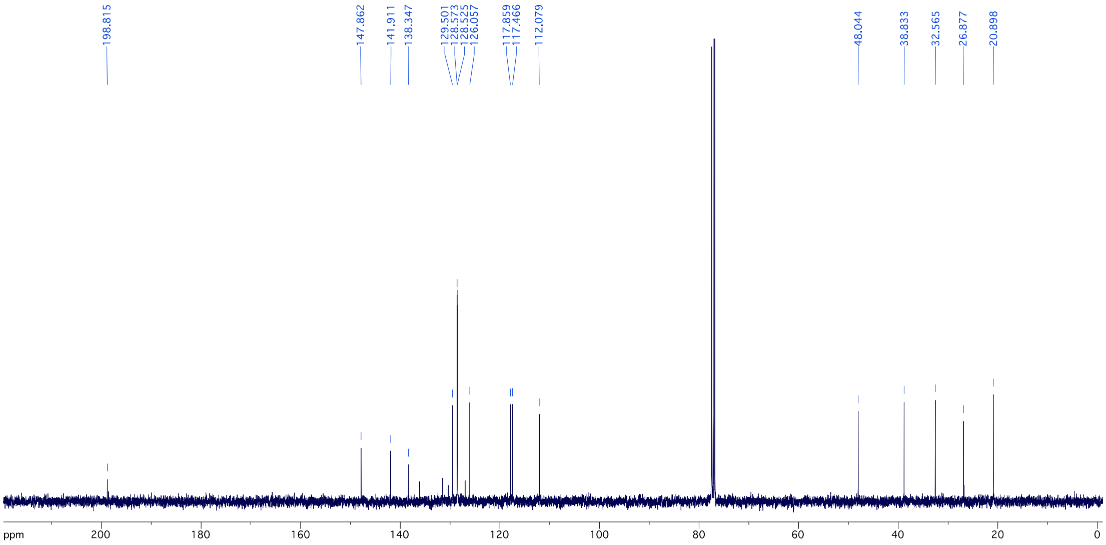

3e – ^{1}H NMR

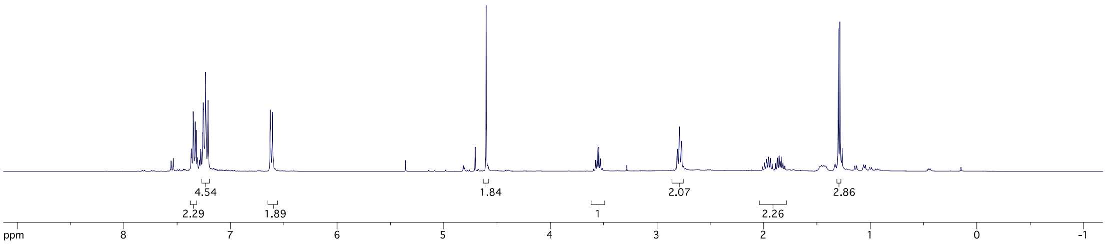

3e – ^{13}C NMR

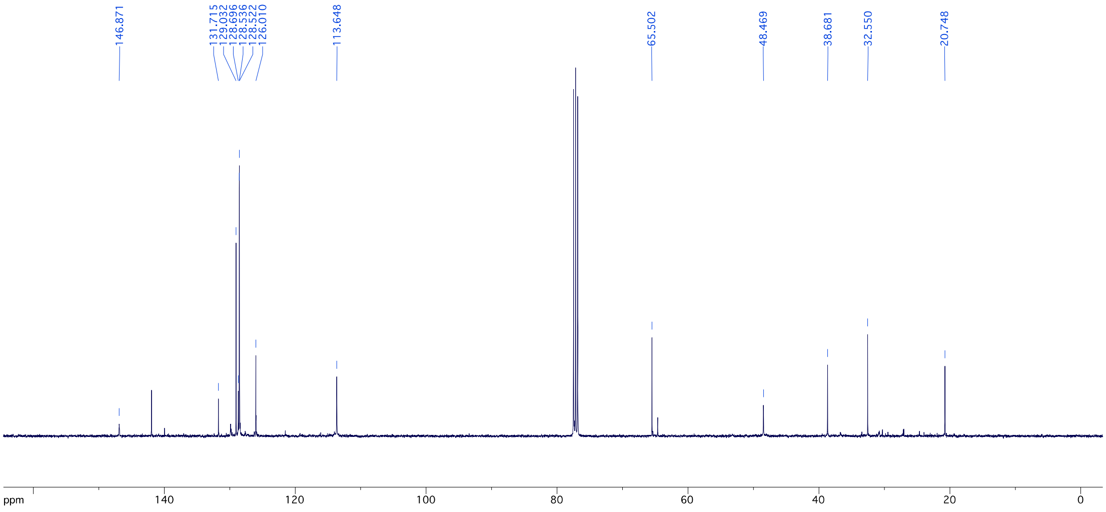

3f – ^{1}H NMR

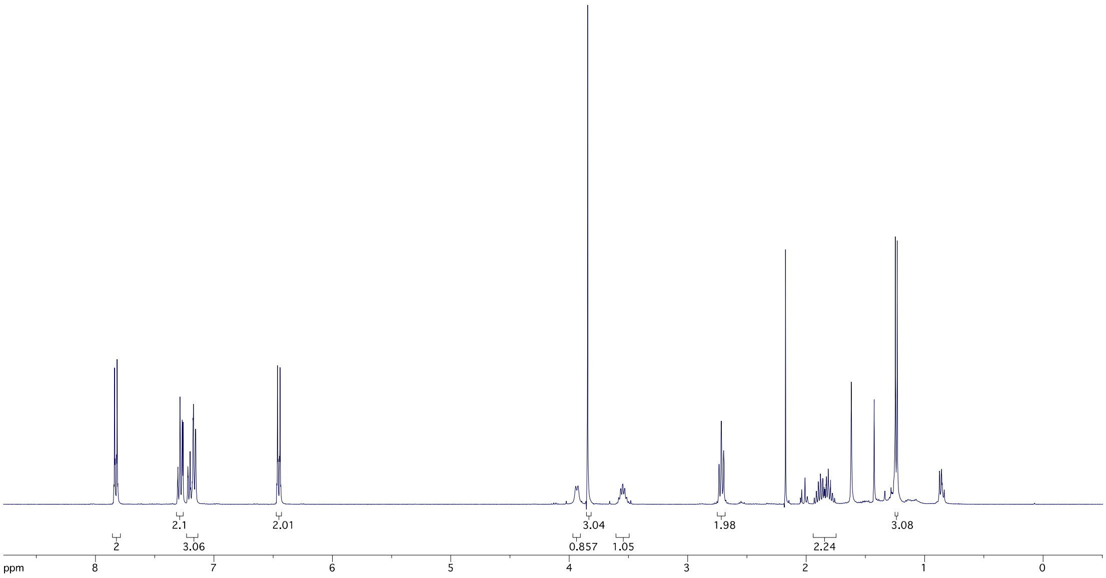

3f – ^{13}C NMR

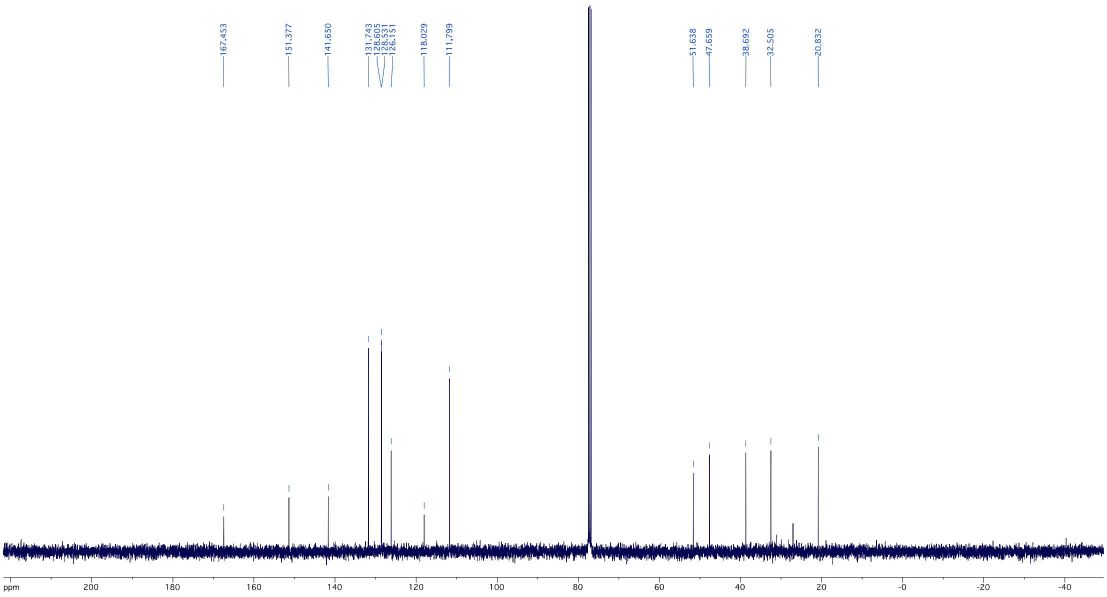

3g – ^{1}H NMR

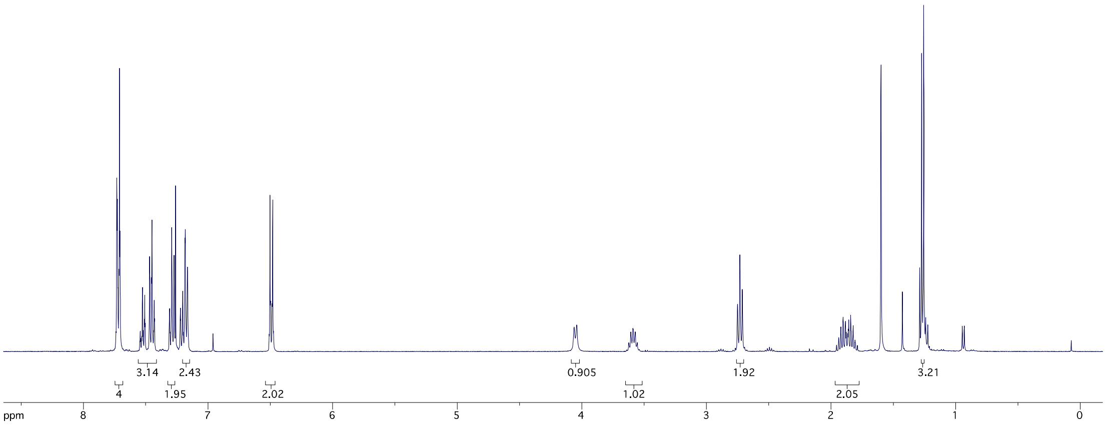

3g – ^{13}C NMR

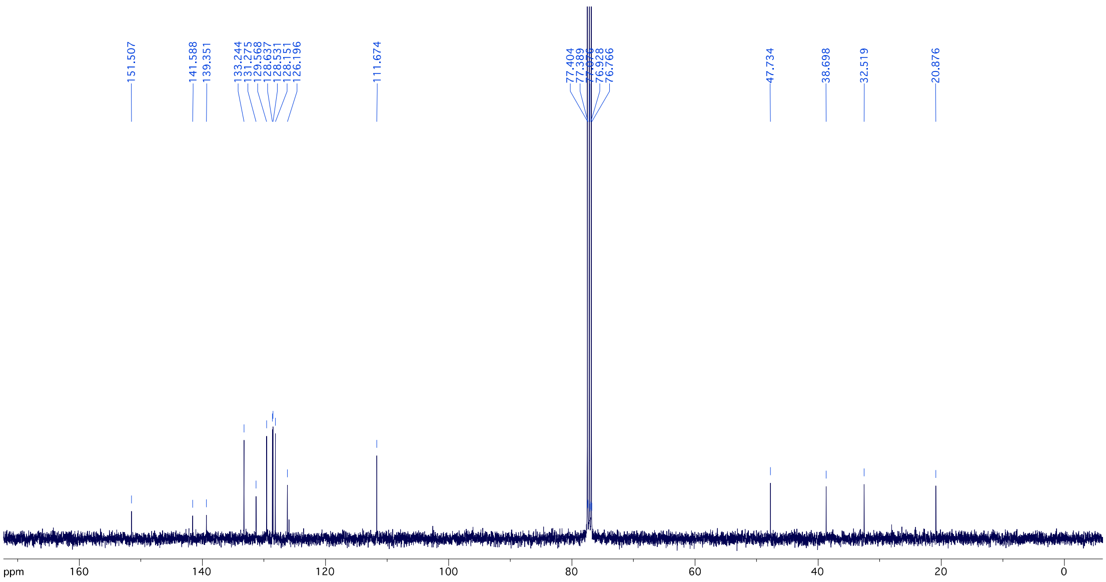

6a – ^{1}H NMR

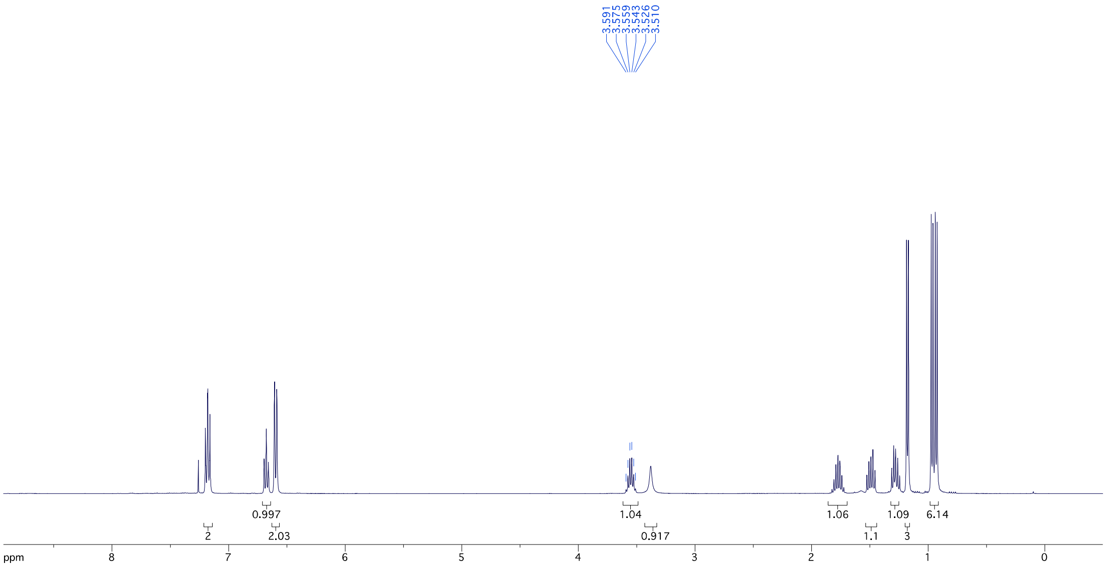

6a – ^{13}C NMR

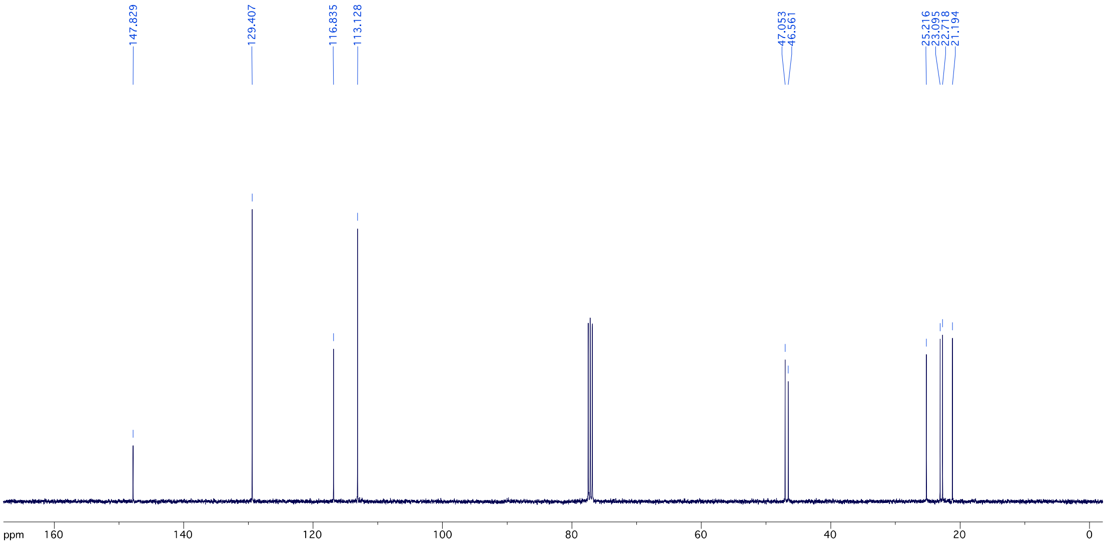

6b – ^{1}H NMR

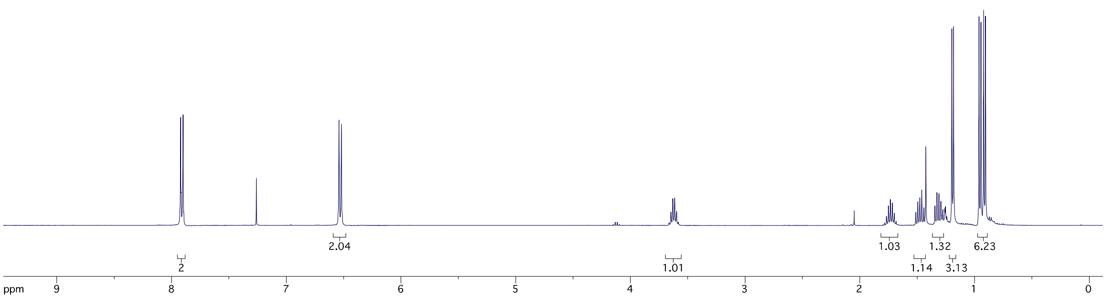

6b – ^{13}C NMR

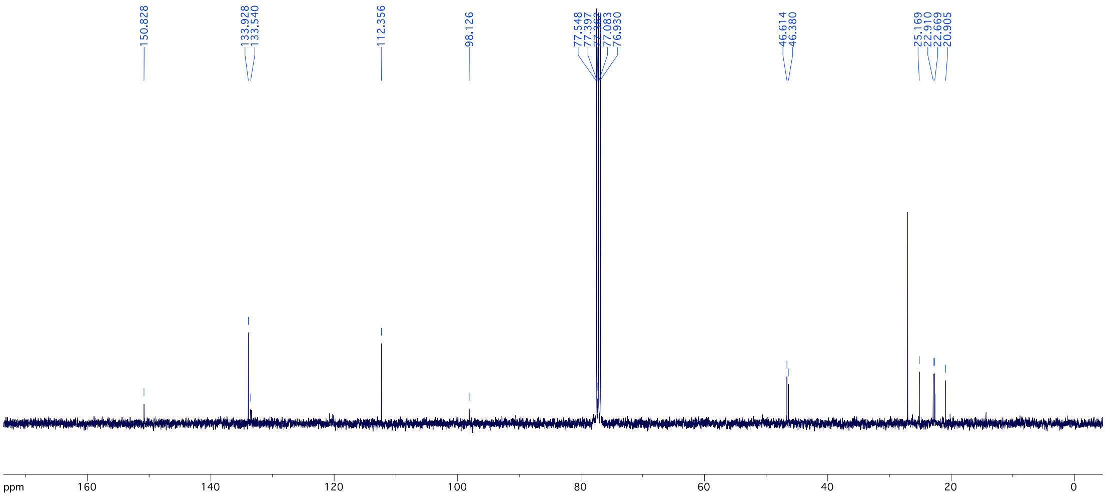

6c – ^{1}H NMR

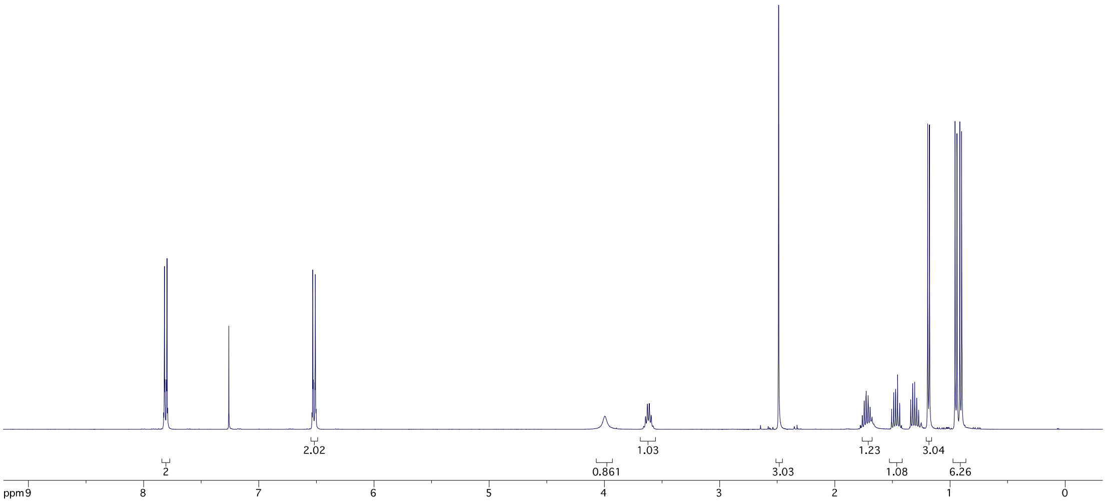

6c – ^{13}C NMR

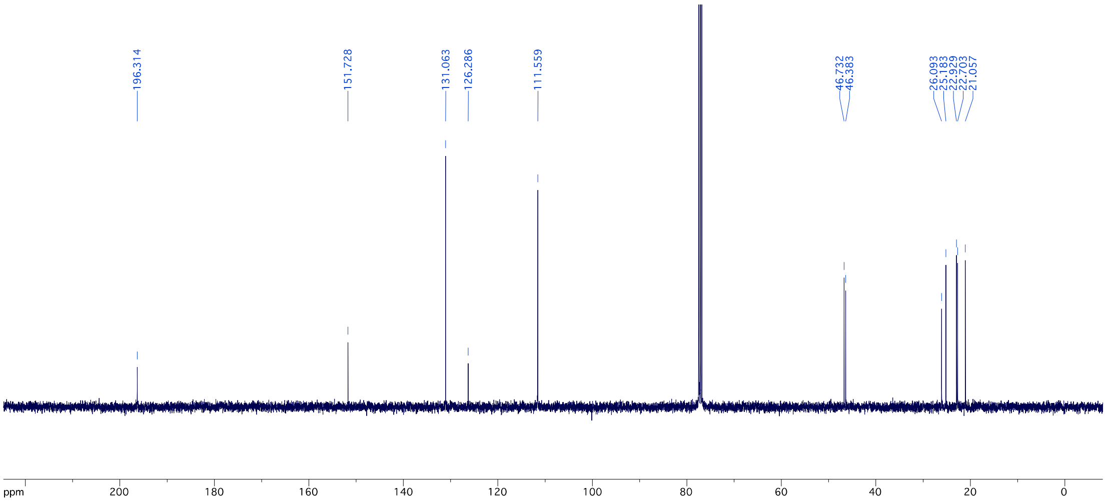

6d – ^{1}H NMR

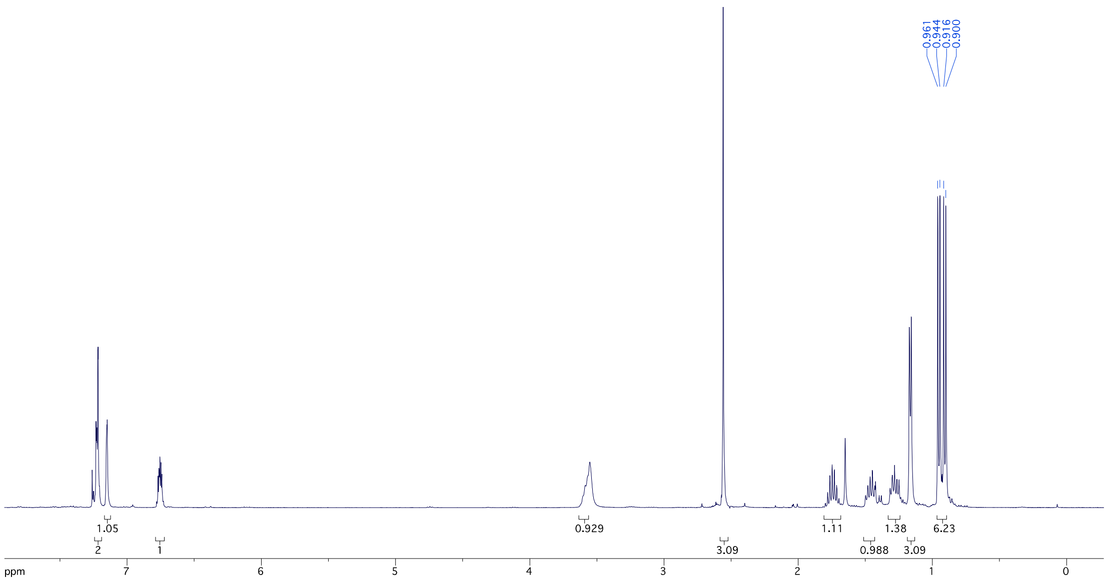

6d – ^{13}C NMR

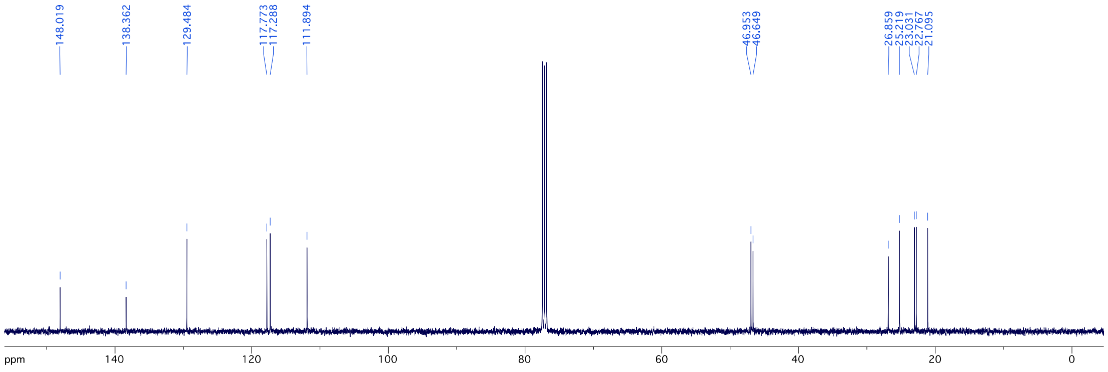

6e – ^{1}H NMR

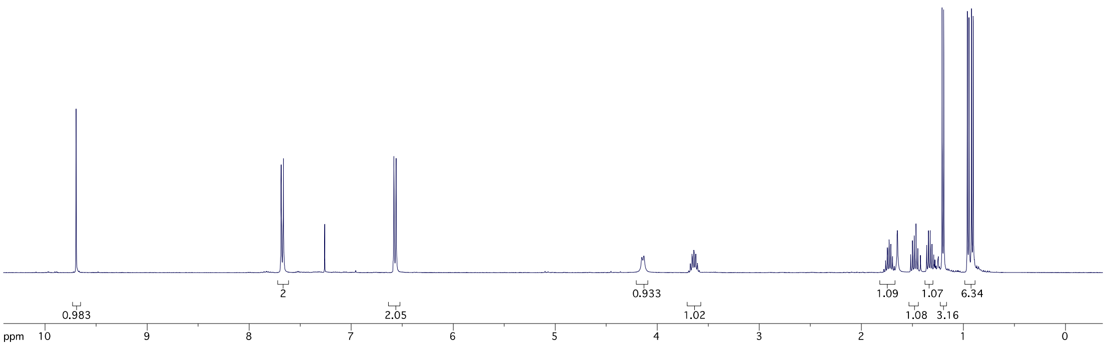

6e – ^{13}C NMR

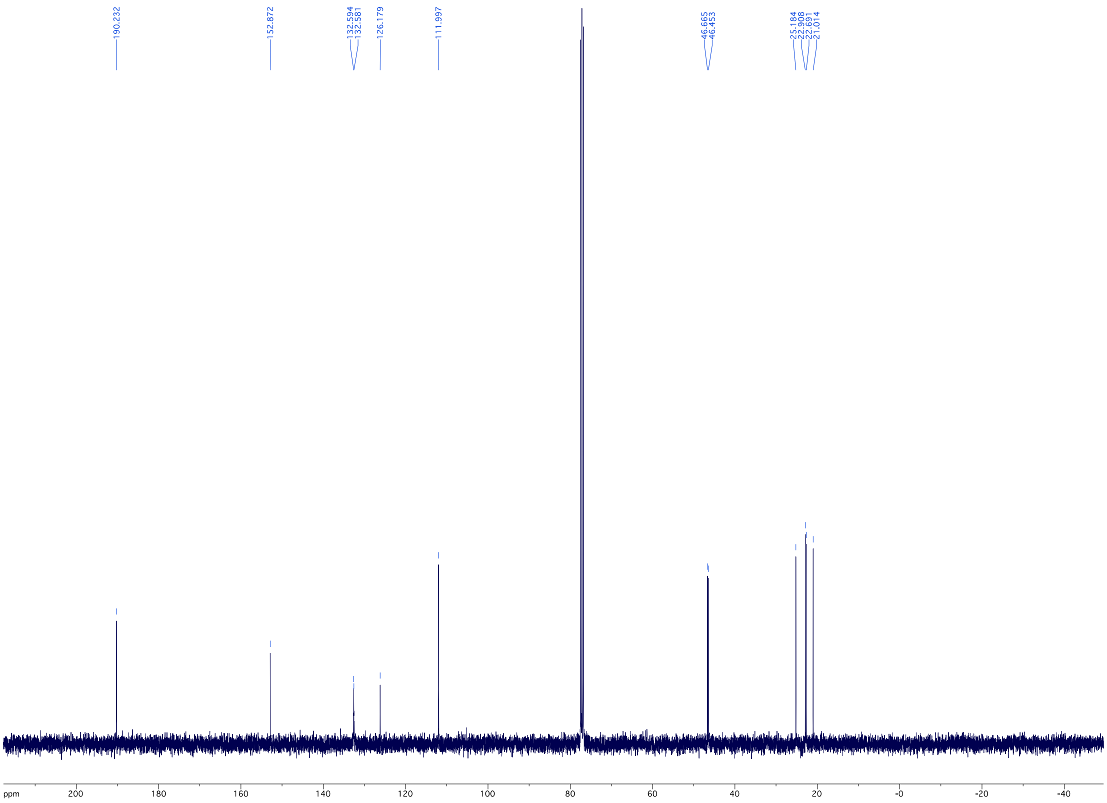

6f – ^{1}H NMR

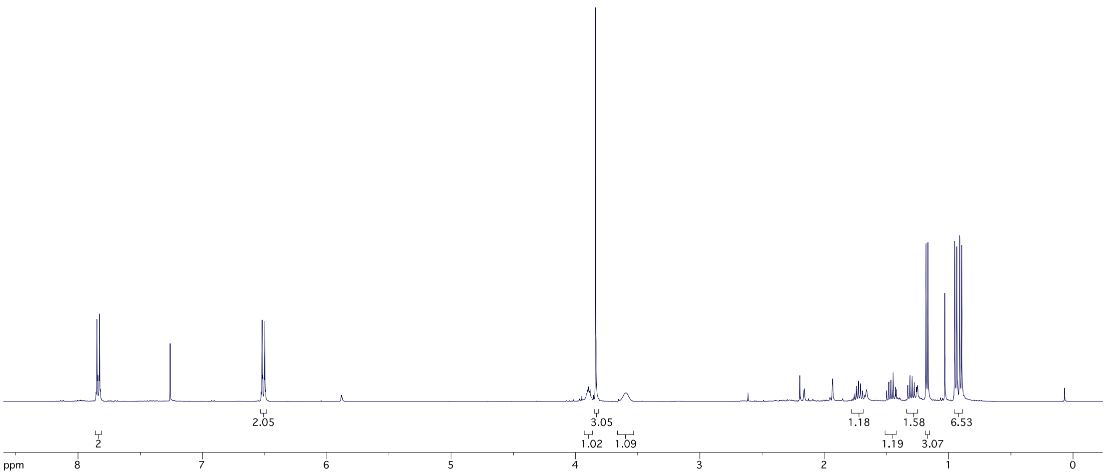

6f – ^{13}C NMR

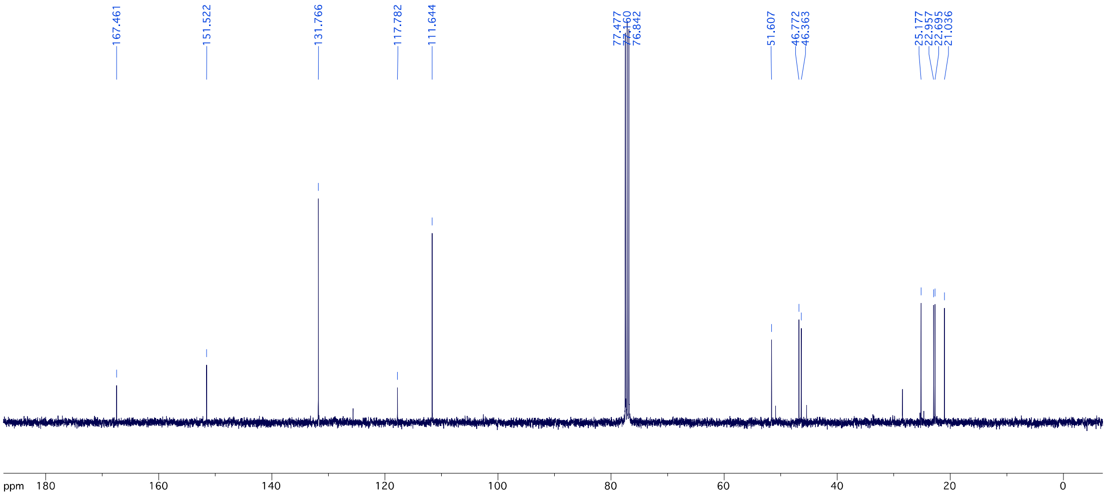

6g – ^{1}H NMR

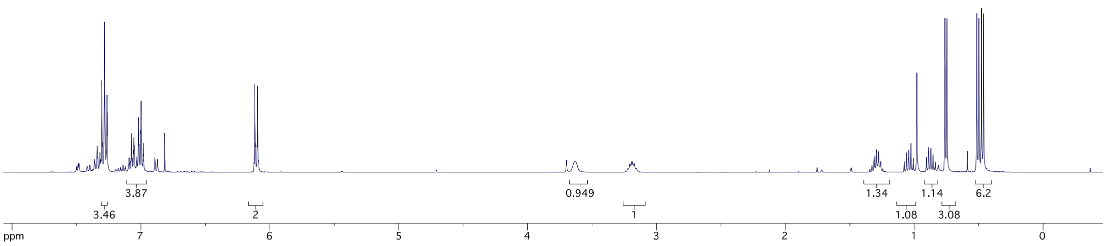

6g – ^{13}C NMR

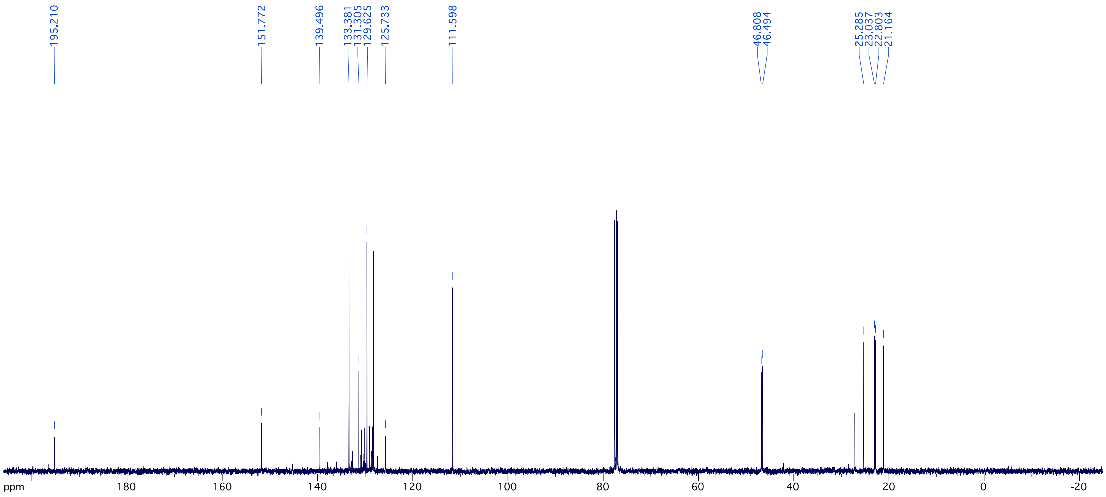

10a – ^{1}H NMR

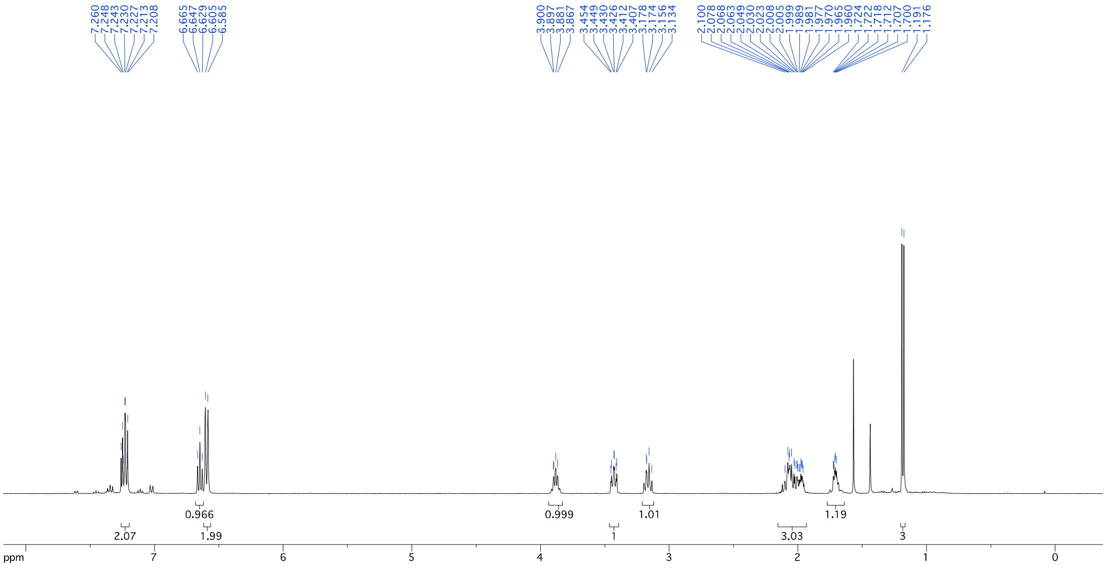

10a – ^{13}C NMR

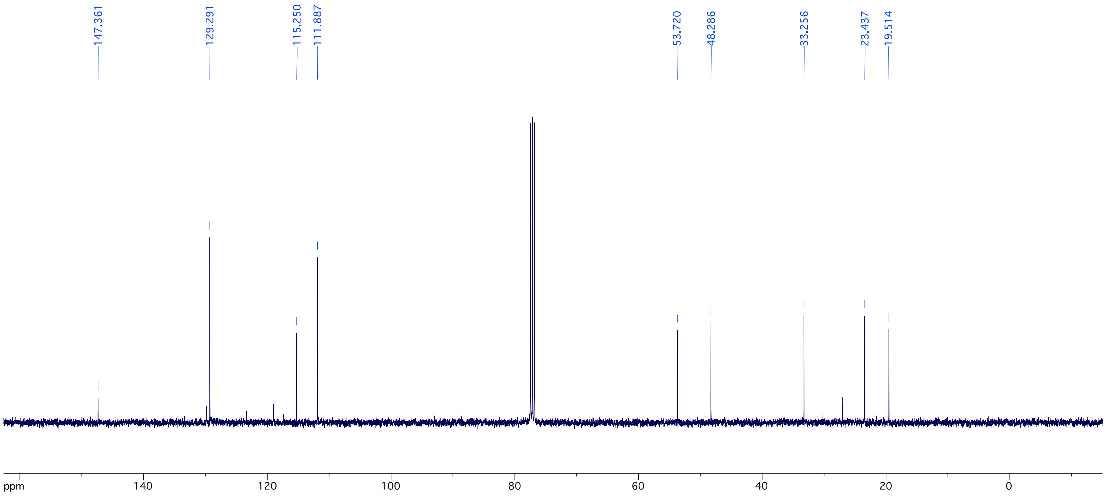

10b – ^{1}H NMR

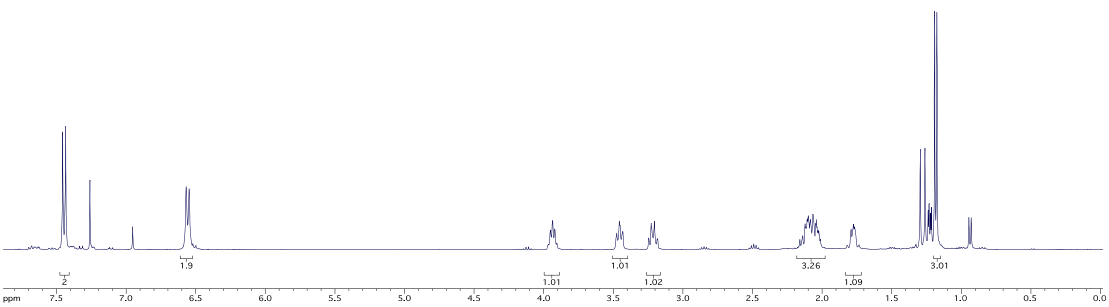

10b – ^{13}C NMR

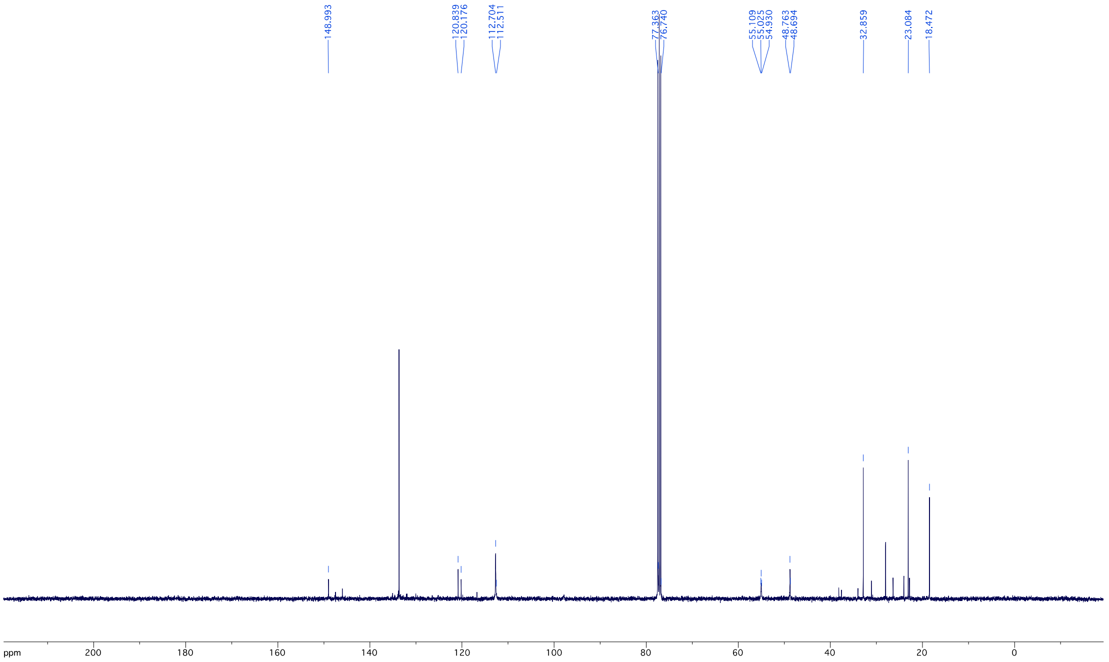

10c – ¹H NMR

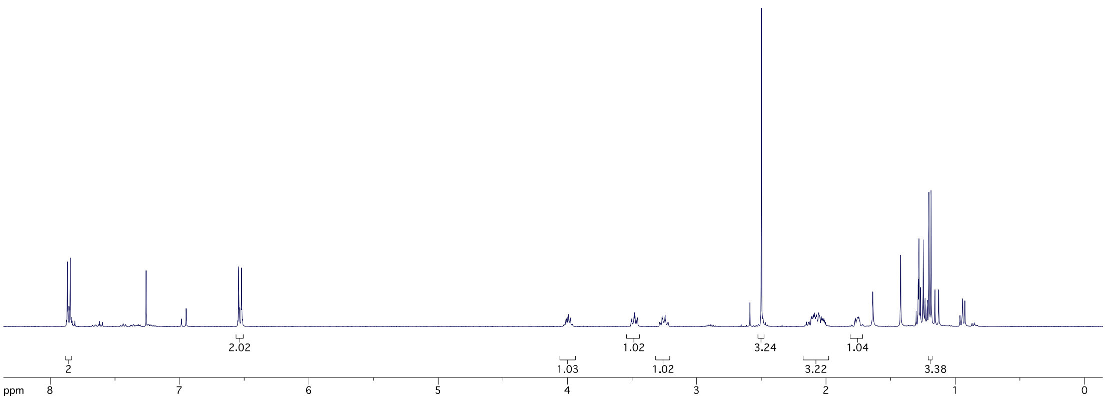

10c – ¹³C NMR

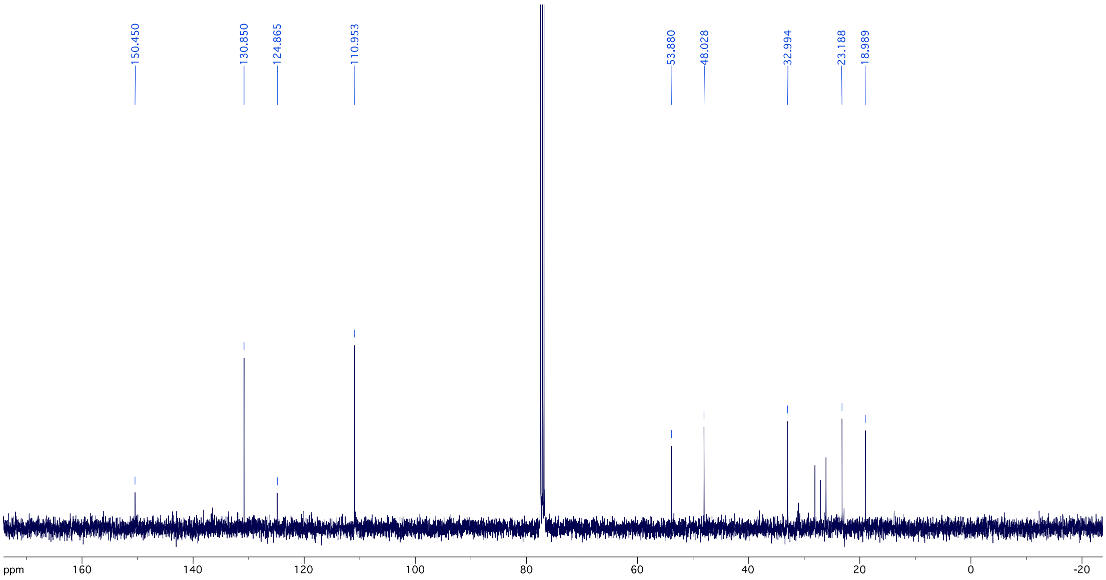

10d – ^{1}H NMR

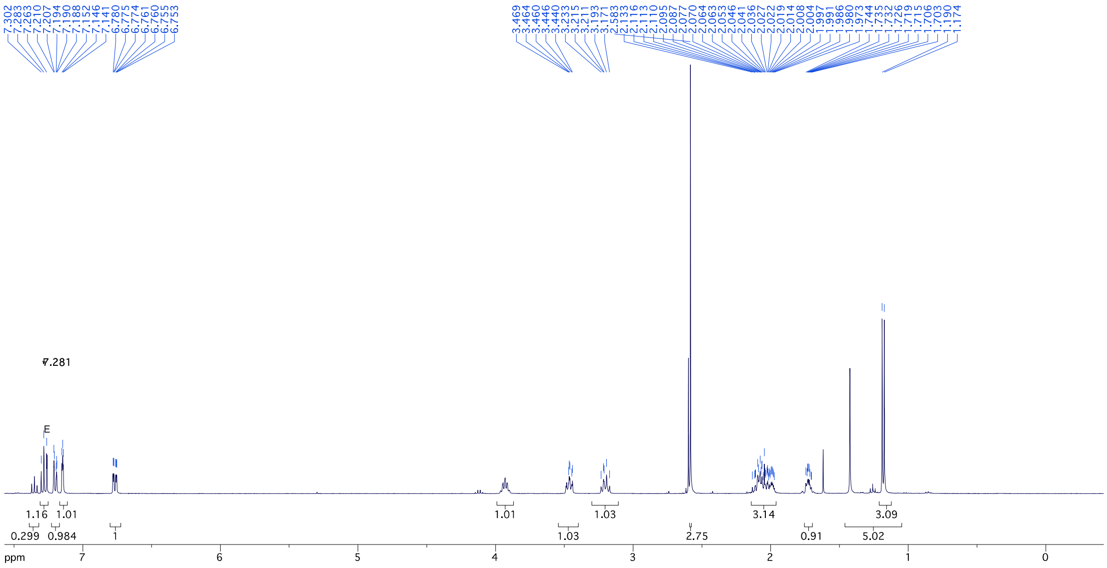

10d – ^{13}C NMR

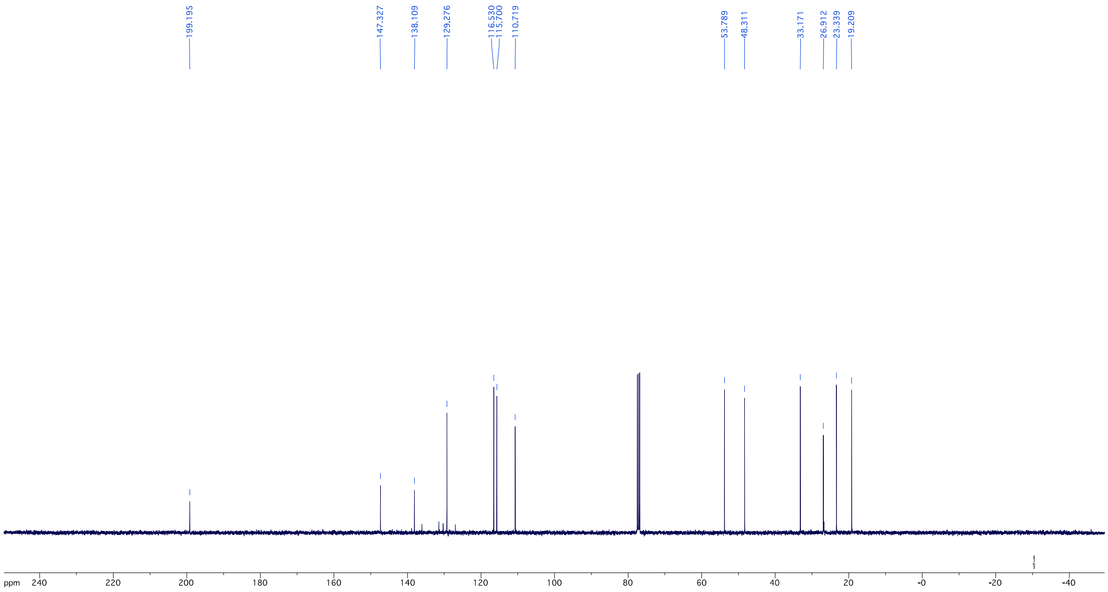

10e – ^{1}H NMR

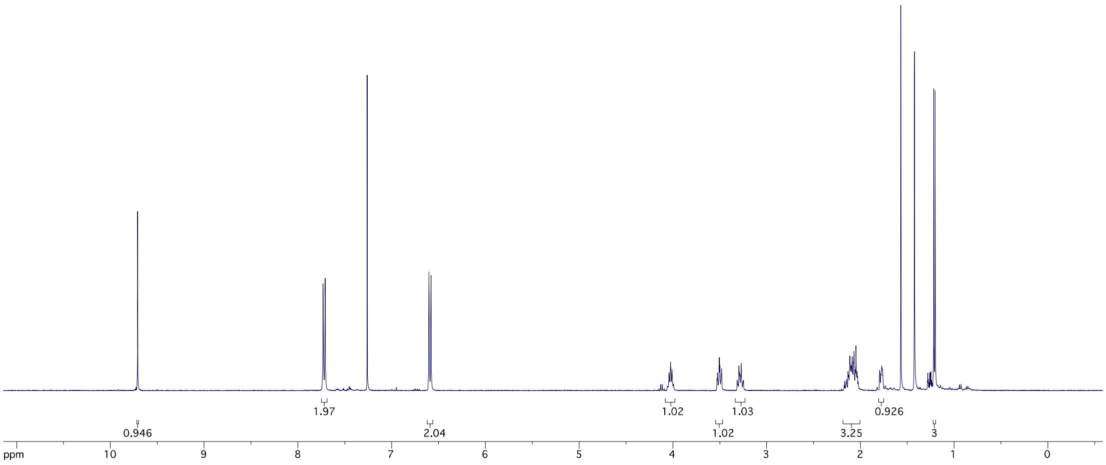

10e – ^{13}C NMR

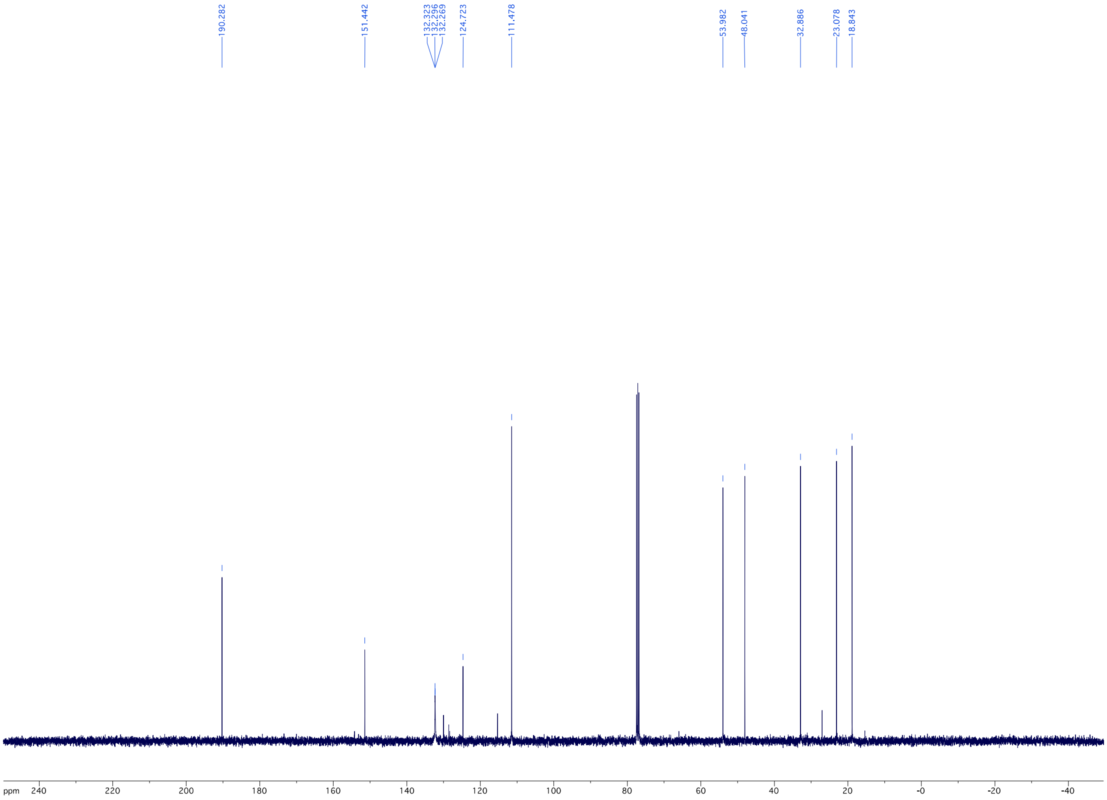

10f – ^{1}H NMR

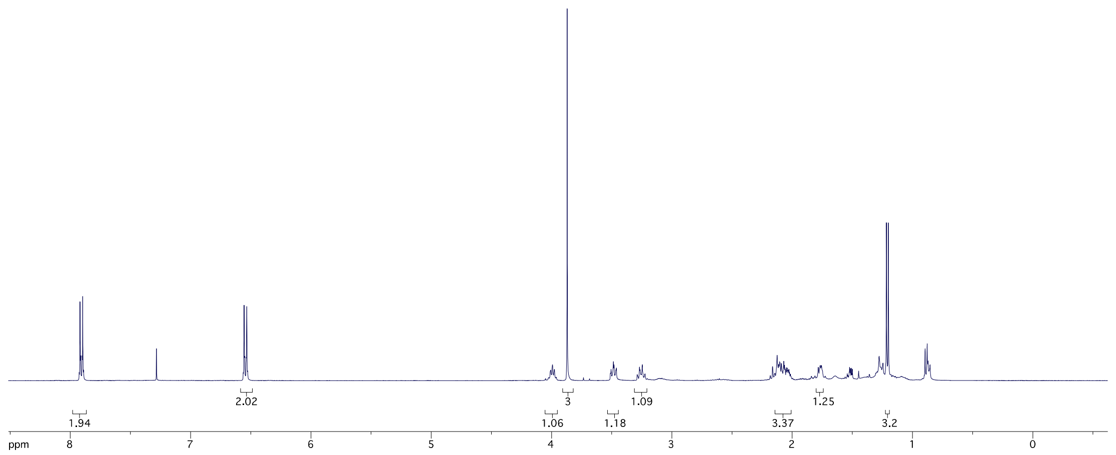

10f – ^{13}C NMR

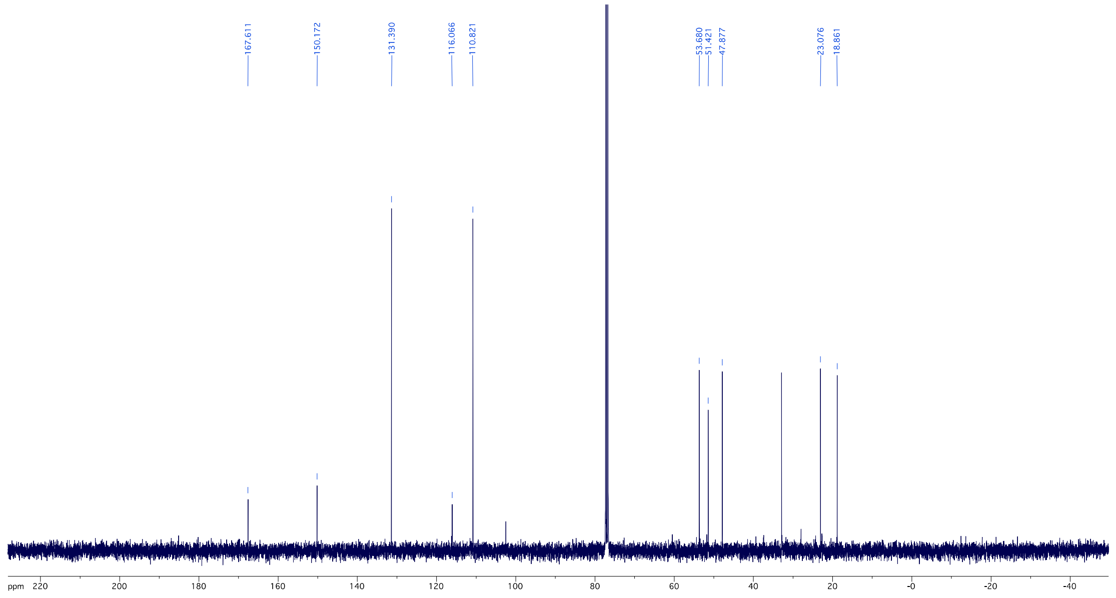

10g – ^{1}H NMR

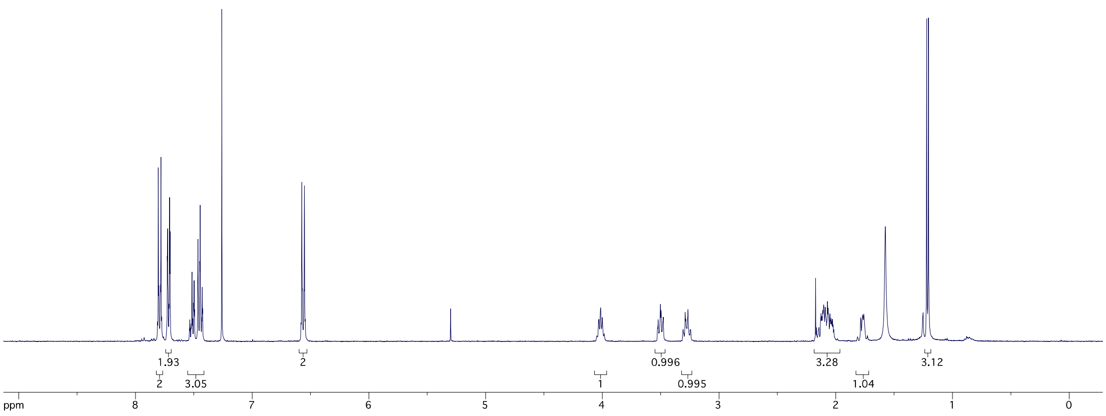

10g – ^{13}C NMR

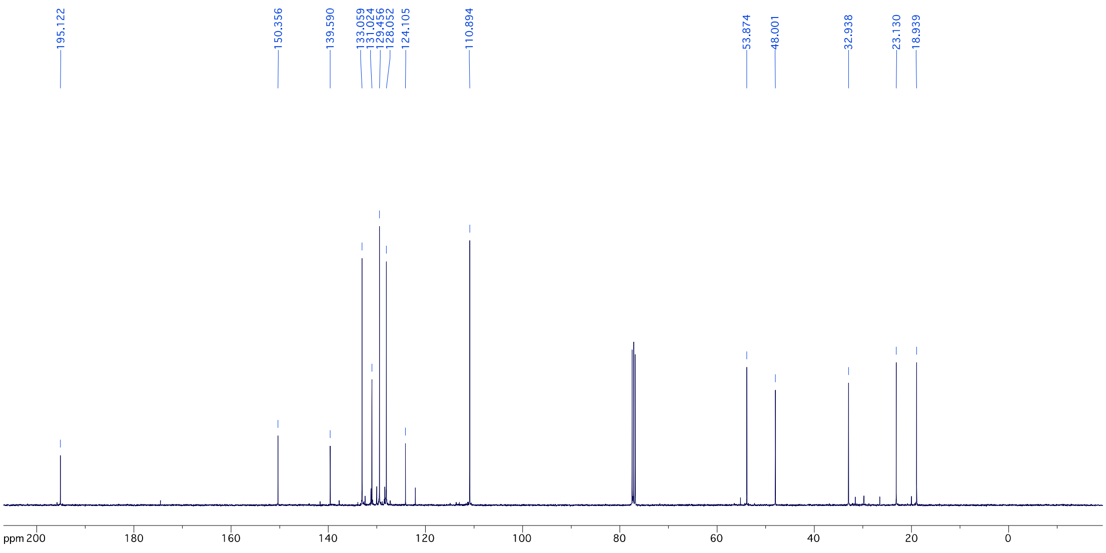

GC Traces

3a

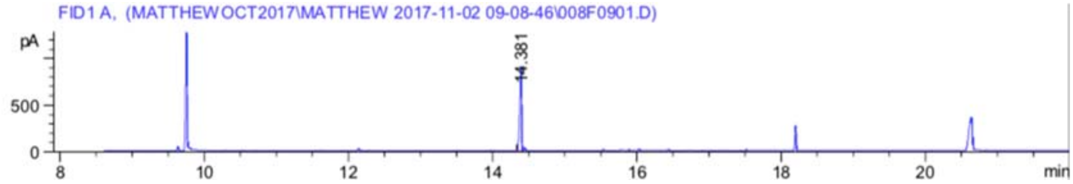

3b

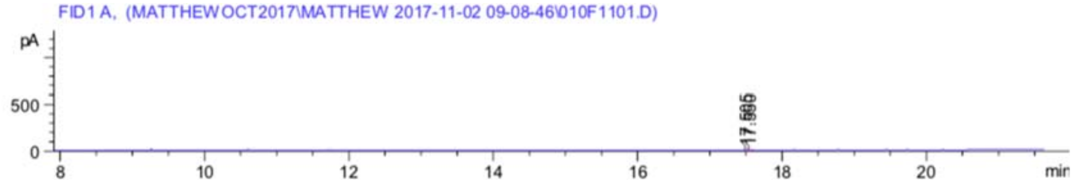

3c

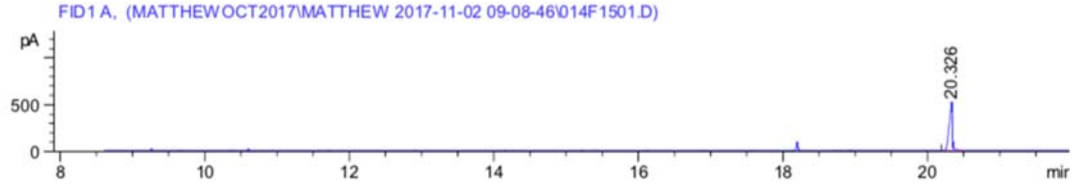

3d

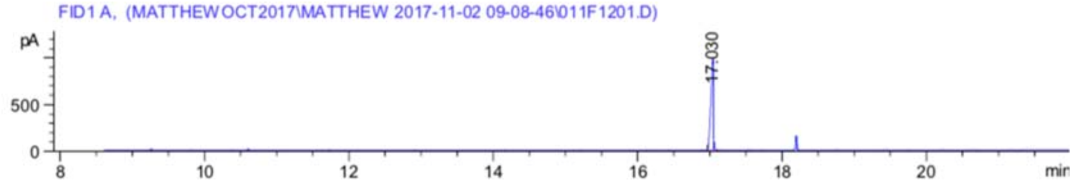

3e

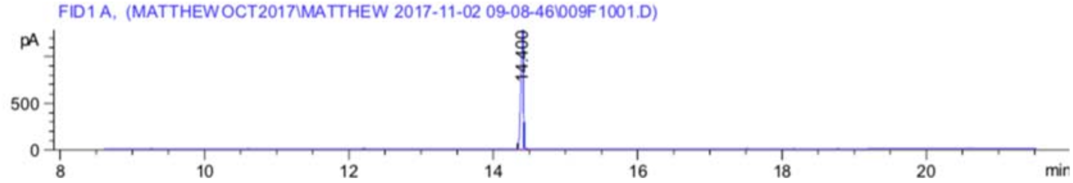

3f

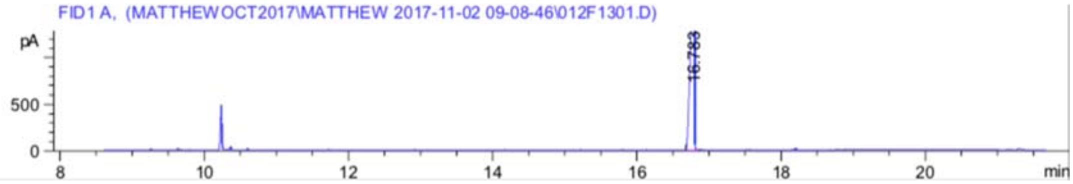

3g

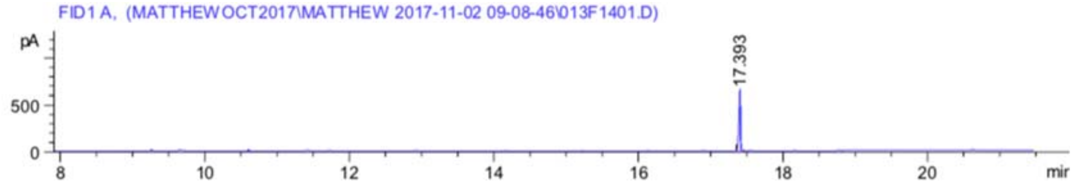

6a

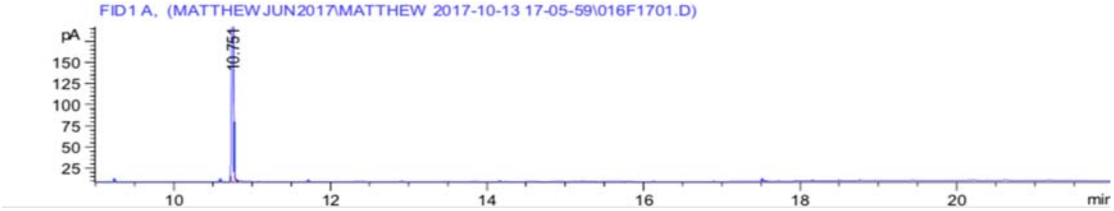

6b

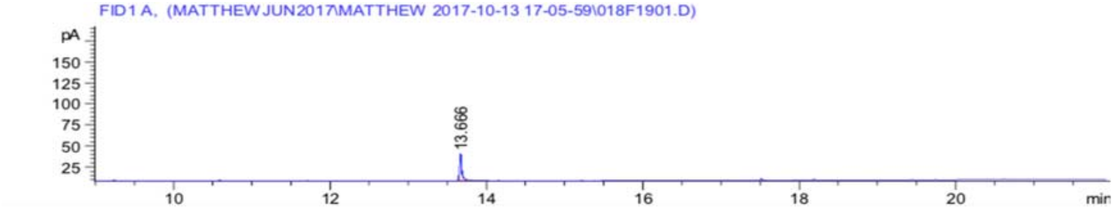

6c

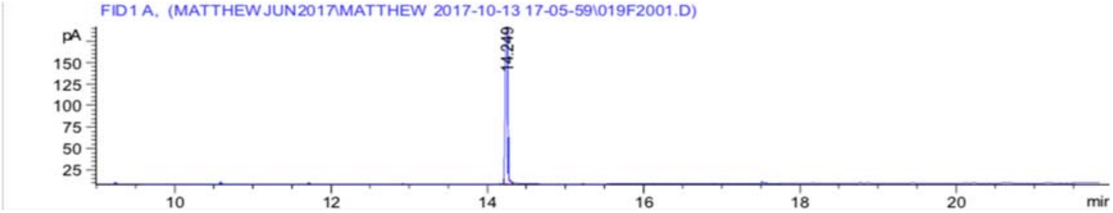

6d

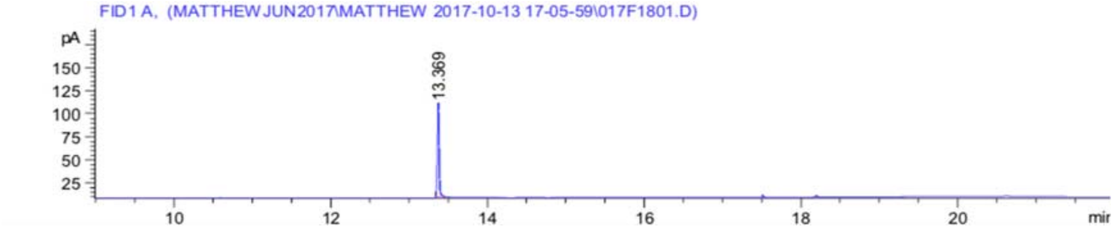

6e

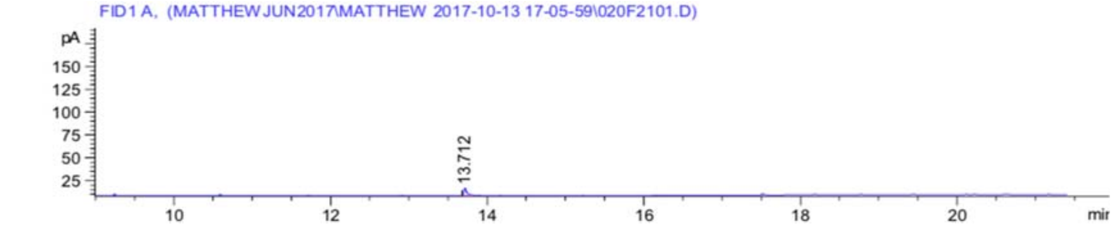

6f

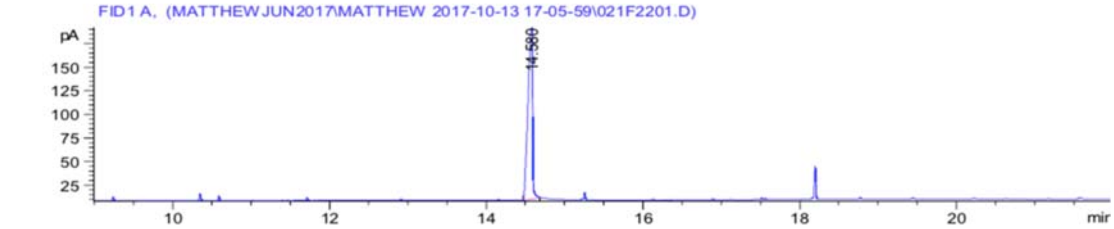

6g

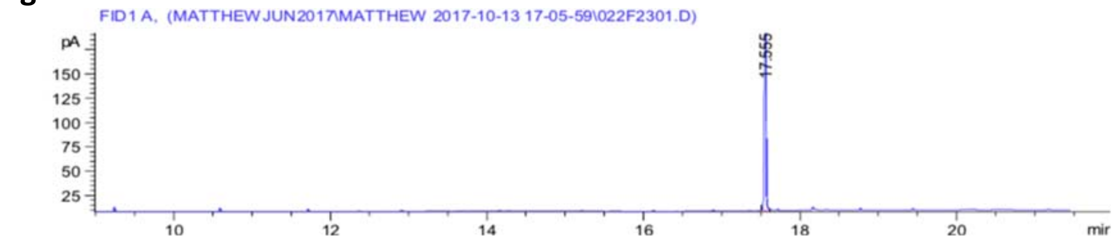

10a

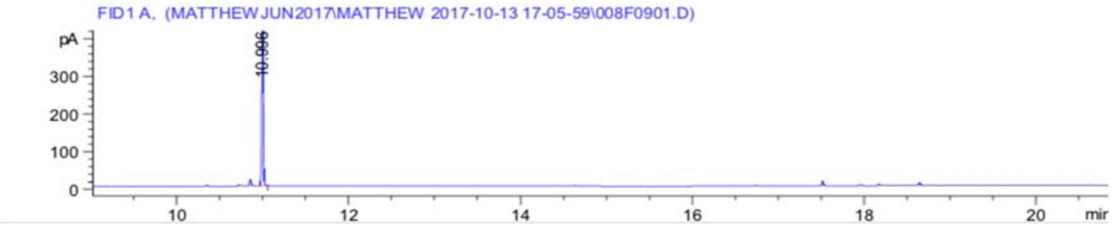

10b

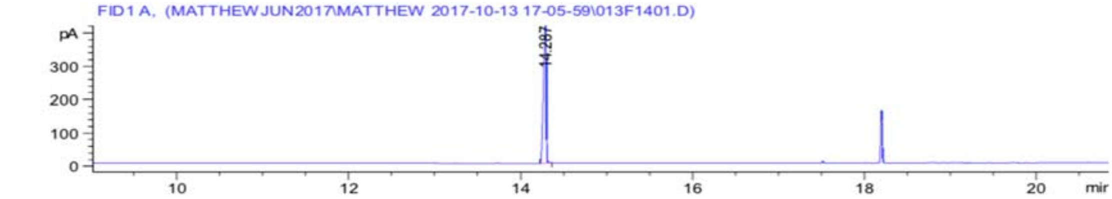

10c

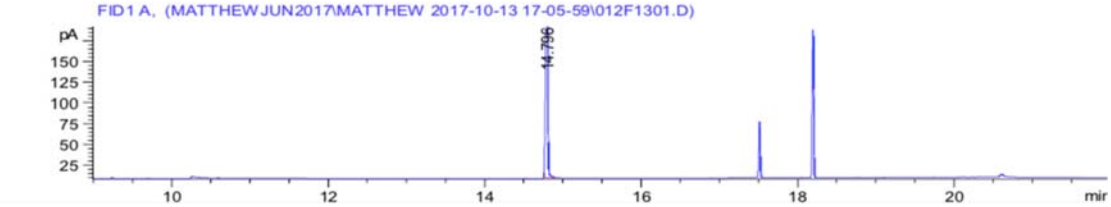

10d

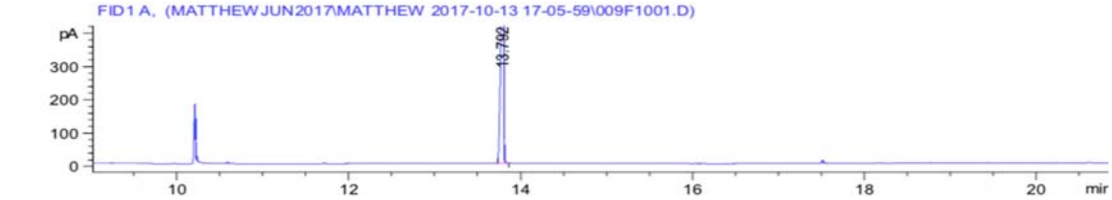

10e

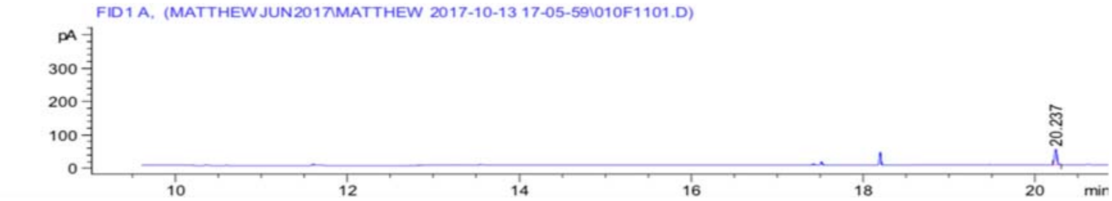

10f

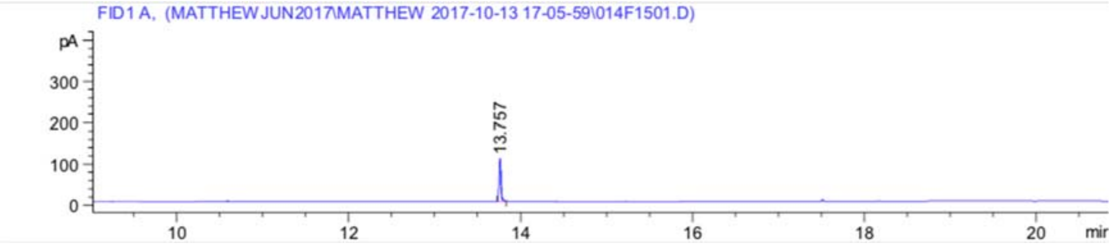

10g

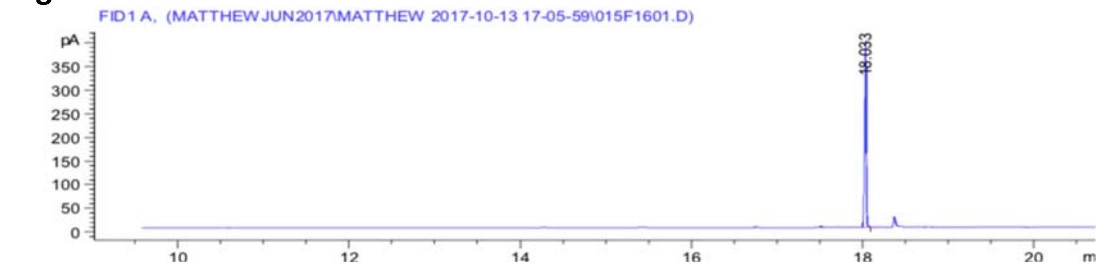

HPLC Traces

3a

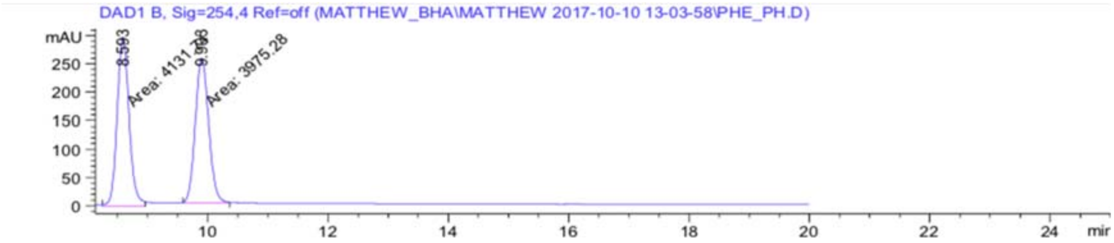

3b

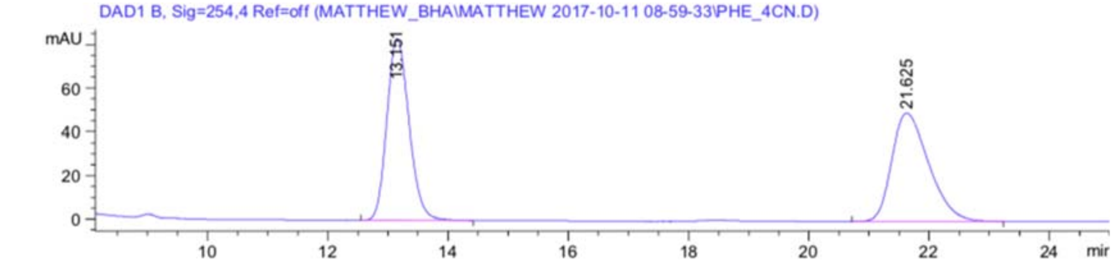

3c

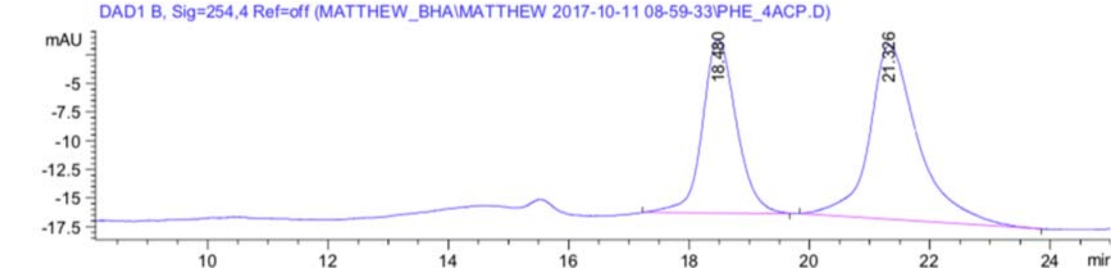

3d

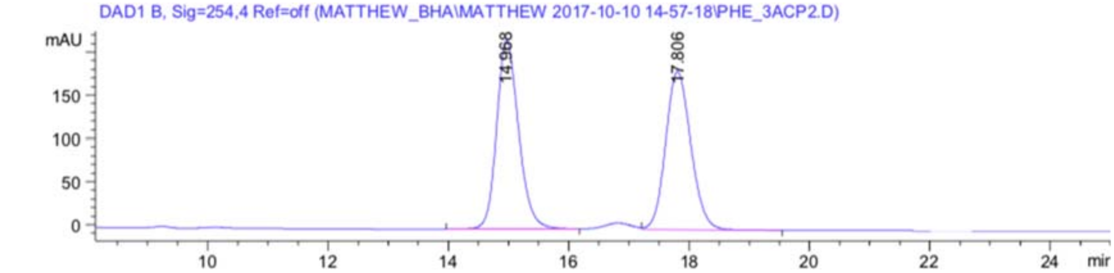

3e

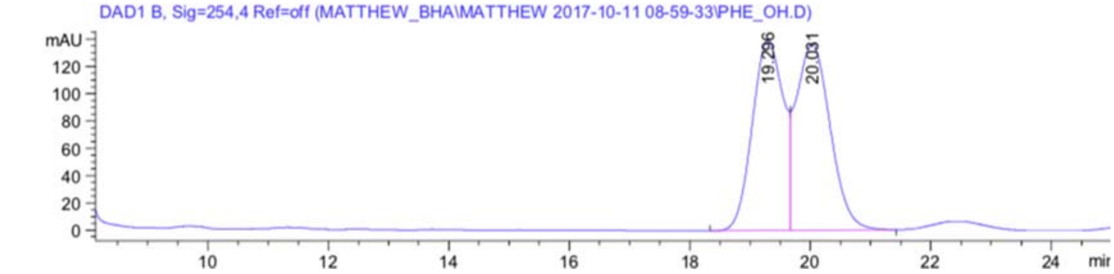

3f

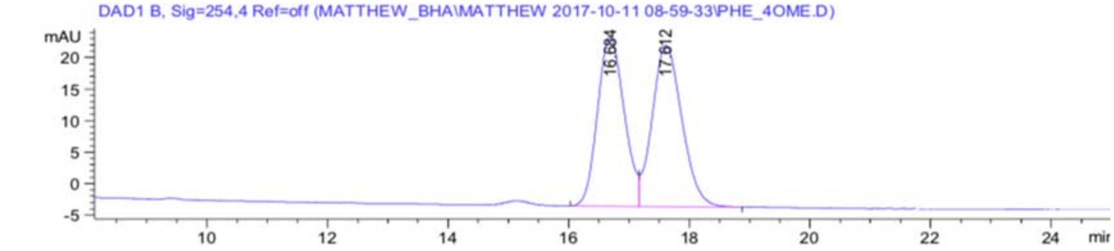

6a

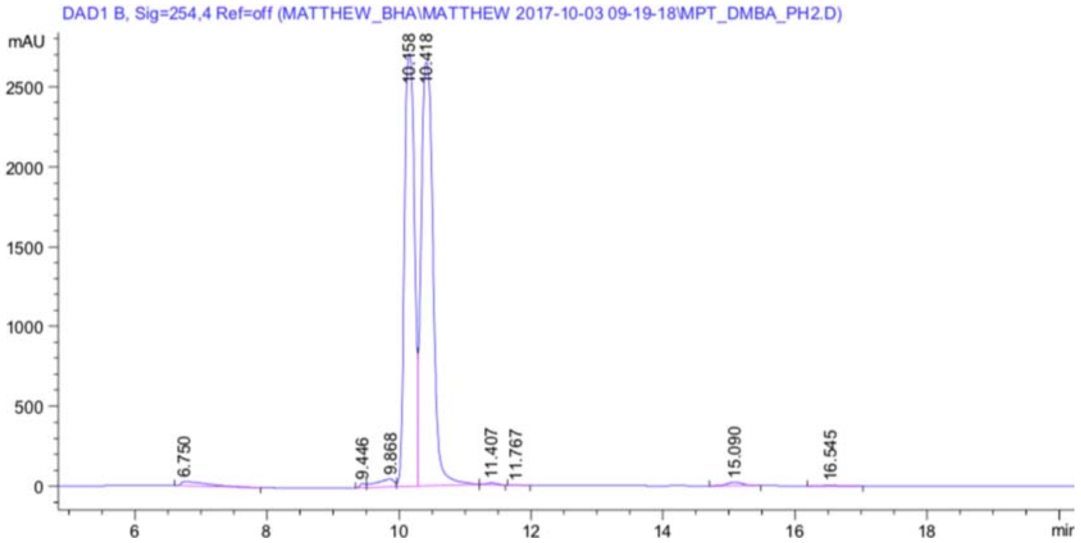

DAD1 B, Sig=254,4 Ref=off (MATTHEW_BHA\MATTHEW 2017-10-03 09-19-18\MPT_DMBA_PH2.D)
mAU
10.158
10.418
6.750
9.446
9.868
11.407
11.767
15.090
16.545

6b

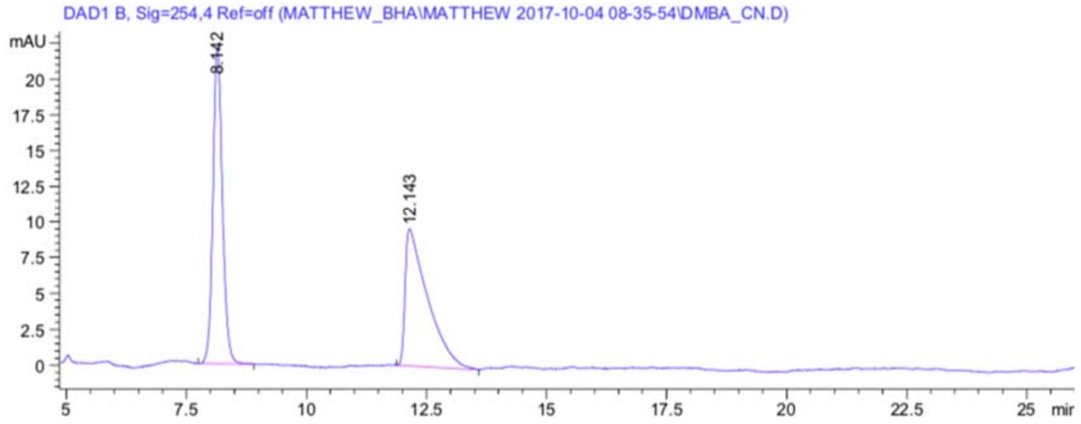

DAD1 B, Sig=254,4 Ref=off (MATTHEW_BHA\MATTHEW 2017-10-04 08-35-54\DMBA_CN.D)
mAU
8.142
12.143

6c

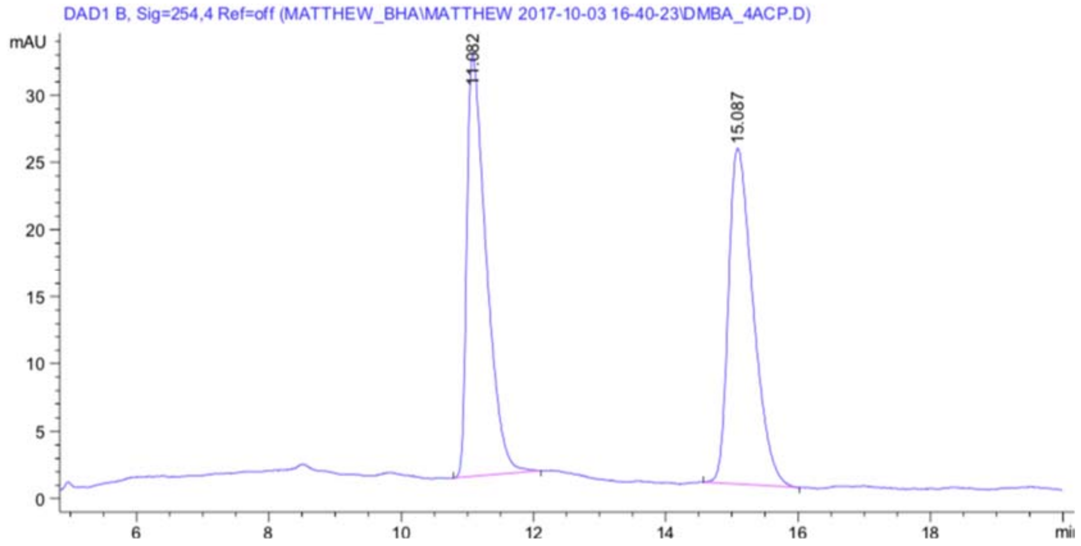

DAD1 B, Sig=254,4 Ref=off (MATTHEW_BHA\MATTHEW 2017-10-03 16-40-23\DMBA_4ACP.D)
mAU
11.082
15.087

6d

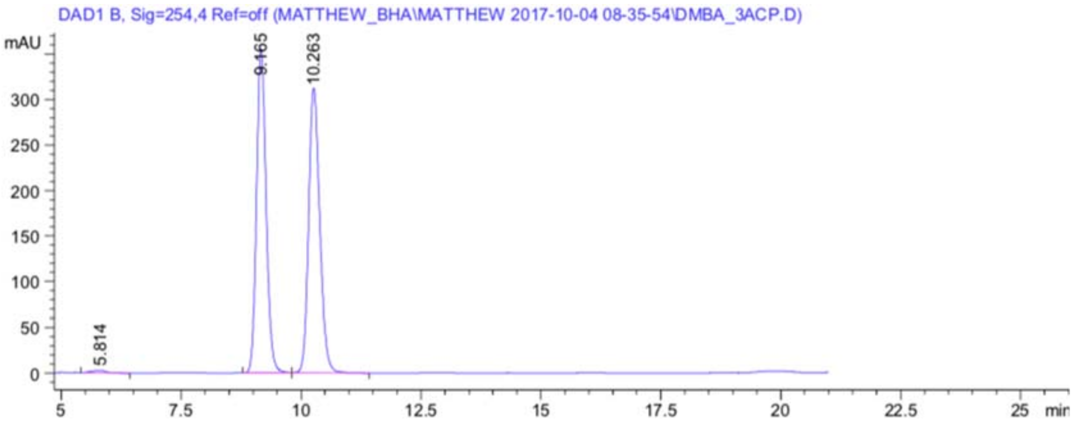
DAD1 B, Sig=254,4 Ref=off (MATTHEW_BHA\MATTHEW 2017-10-04 08-35-54\DMBA_3ACP.D)
mAU
5.814
9.165
10.263
min

6e

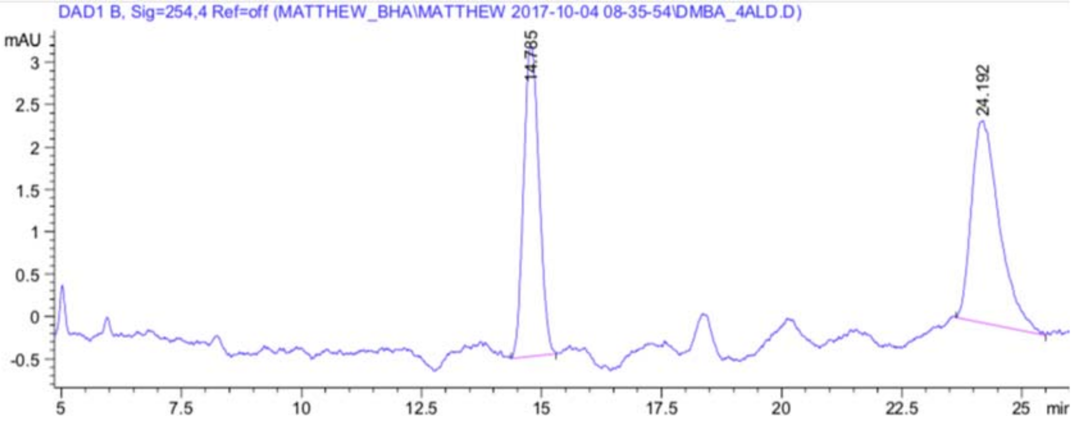
DAD1 B, Sig=254,4 Ref=off (MATTHEW_BHA\MATTHEW 2017-10-04 08-35-54\DMBA_4ALD.D)
mAU
14.785
24.192
min

6f

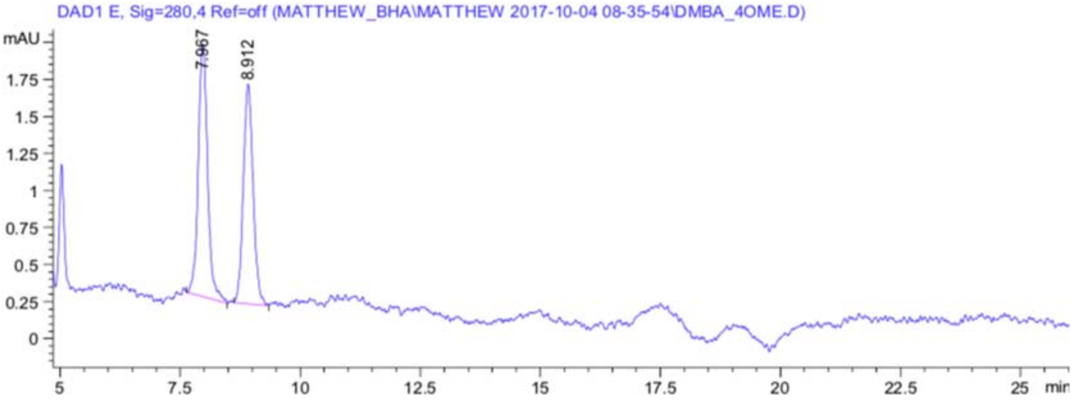
DAD1 E, Sig=280,4 Ref=off (MATTHEW_BHA\MATTHEW 2017-10-04 08-35-54\DMBA_4OME.D)
mAU
7.967
8.912
min

6g

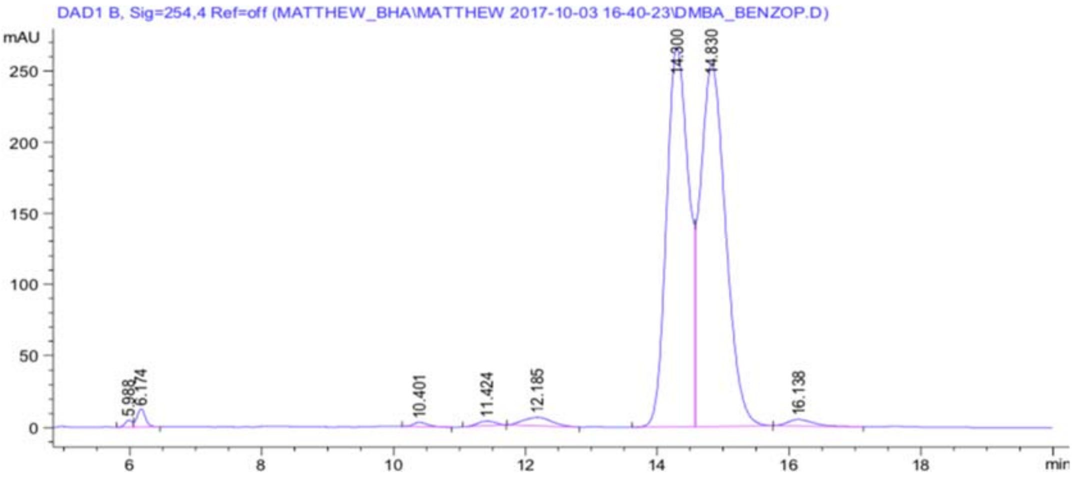
DAD1 B, Sig=254,4 Ref=off (MATTHEW_BHA\MATTHEW 2017-10-03 16-40-23\DMBA_BENZOP.D)
mAU
5.988
6.174
10.401
11.424
12.185
14.300
14.830
16.138
min

10c

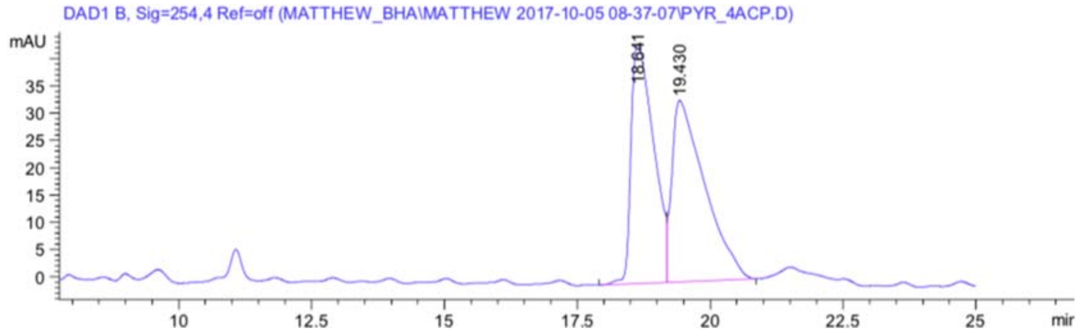

10d

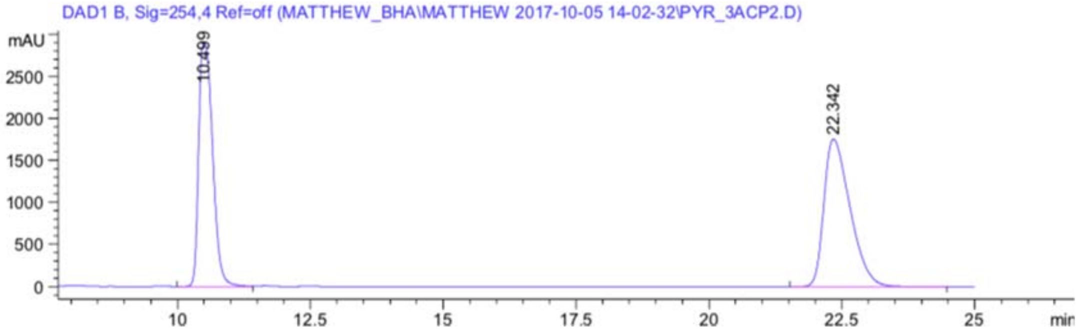

10e

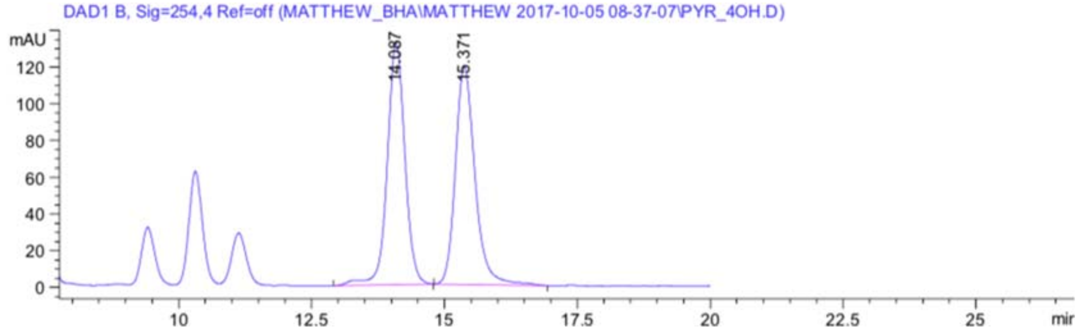

10f

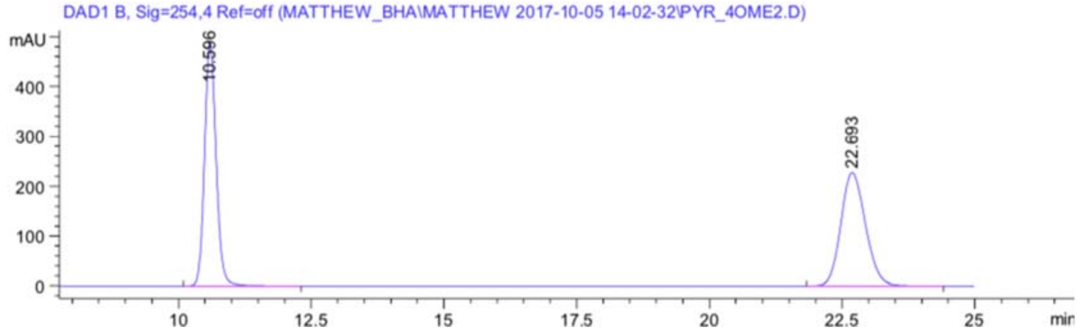

10g

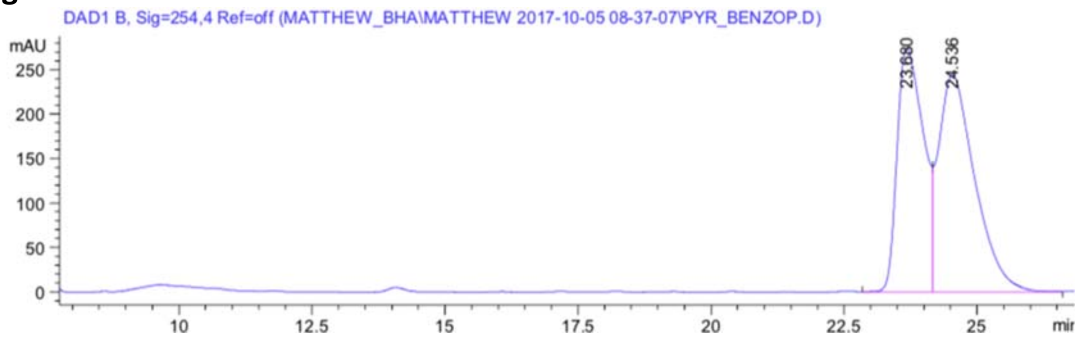

6c - ^{1}H NMR from scaled up amination of 11

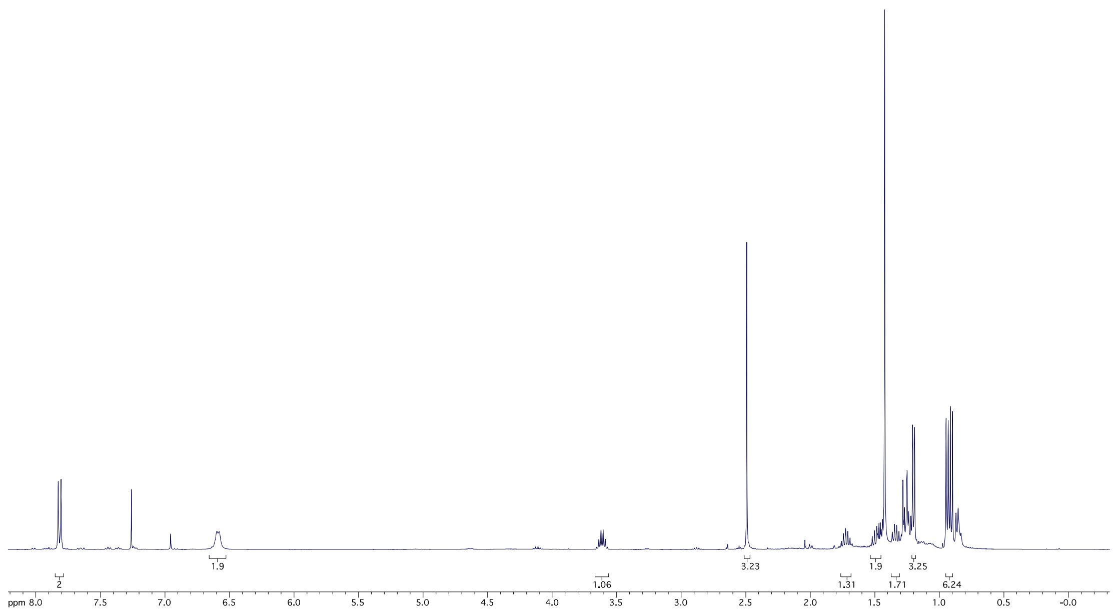

^{1}H and ^{13}C NMR spectra and GC traces of preparative-scale arylation of 10a

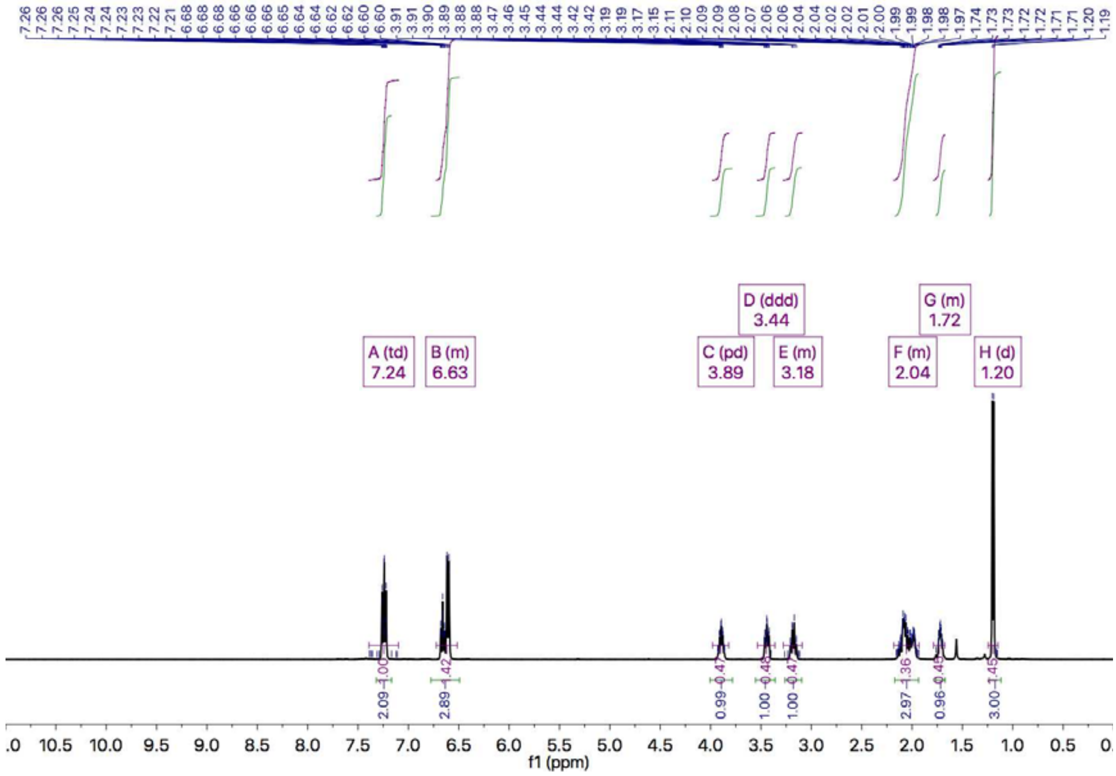

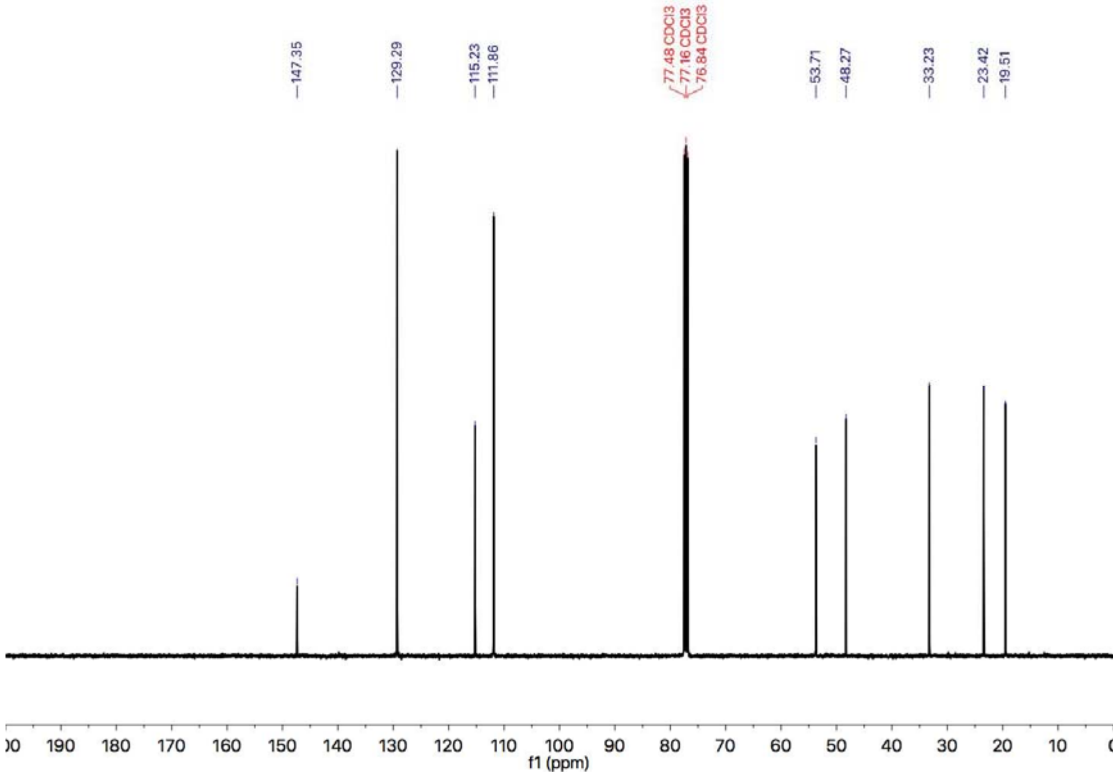

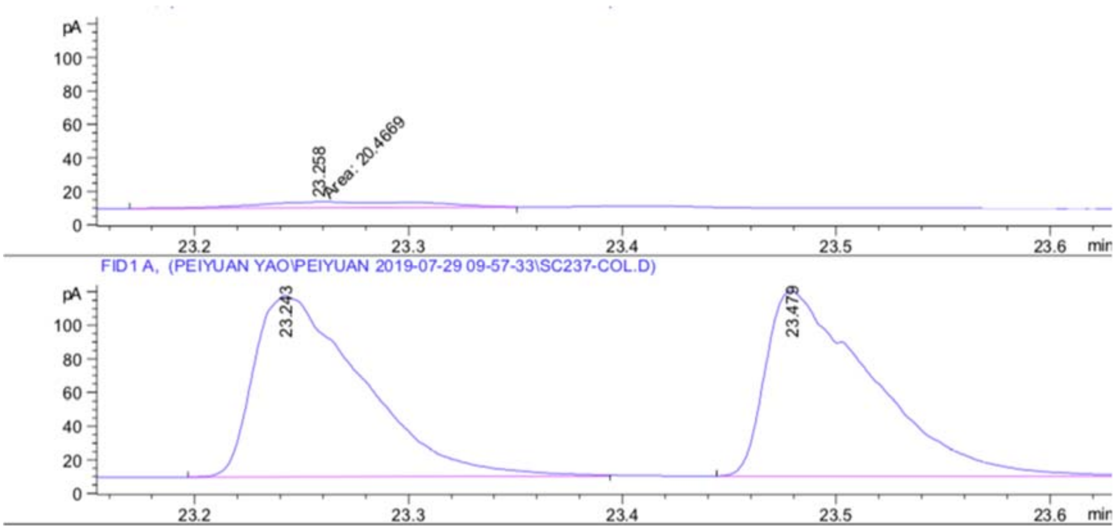

pA
100
80
60
40
20
0
23.258
Area: 20.4669
23.2
23.3
23.4
23.5
23.6
min
FID1 A, (PEIYUAN YAO\PEIYUAN 2019-07-29 09-57-33\SC237-COL.D)
pA
100
80
60
40
20
0
23.243
23.479
23.2
23.3
23.4
23.5
23.6
min

References

[1] M. P. Thompson, N. J. Turner, *ChemCatChem* **2017**, *9*, 3833–3836.

[2] T. Knaus, W. Böhmer, F. G. Mutti, *Green Chem.* **2017**, *19*, 453–463.

[3] H. Man, E. Wells, S. Hussain, F. Leipold, S. Hart, J. P. Turkenburg, N. J. Turner, G. Grogan, *ChemBioChem* **2015**, 1052–1059.
